# BMAL1/p53 mediating bronchial epithelial cell autophagy contributes to PM2.5-aggravated asthma

**DOI:** 10.1186/s12964-023-01057-9

**Published:** 2023-02-20

**Authors:** Shuai-Jun Chen, Yi Huang, Fan Yu, Xiao Feng, Yuan-Yi Zheng, Qian Li, Qian Niu, Ye-Han Jiang, Li-Qin Zhao, Meng Wang, Pei-Pei Cheng, Lin-Jie Song, Li-Mei Liang, Xin-Liang He, Liang Xiong, Fei Xiang, Xiaorong Wang, Wan-Li Ma, Hong Ye

**Affiliations:** 1 Department of Pathophysiology, School of Basic Medicine, Tongji Medical College, Huazhong University of Science and Technology, 13 Hang Kong Road, Wuhan, 430030 China; 2 Department of Respiratory and Critical Care Medicine, Union Hospital, Tongji Medical College, Huazhong University of Science and Technology, 1277 JieFang Avenue, Wuhan, 430022 China; 3 Key Laboratory of Respiratory Diseases, National Health Commission of China, Wuhan, China

**Keywords:** PM2.5, Asthma, Remodeling, BMAL1, p53, Autophagy, Bronchial epithelial cells

## Abstract

**Background:**

Fine particulate matter (PM2.5) is associated with increased incidence and severity of asthma. PM2.5 exposure disrupts airway epithelial cells, which elicits and sustains PM2.5-induced airway inflammation and remodeling. However, the mechanisms underlying development and exacerbation of PM2.5-induced asthma were still poorly understood. The aryl hydrocarbon receptor nuclear translocator-like protein 1 (BMAL1) is a major circadian clock transcriptional activator that is also extensively expressed in peripheral tissues and plays a crucial role in organ and tissue metabolism.

**Results:**

In this study, we found PM2.5 aggravated airway remodeling in mouse chronic asthma, and exacerbated asthma manifestation in mouse acute asthma. Next, low BMAL1 expression was found to be crucial for airway remodeling in PM2.5-challenged asthmatic mice. Subsequently, we confirmed that BMAL1 could bind and promote ubiquitination of p53, which can regulate p53 degradation and block its increase under normal conditions. However, PM2.5-induced BMAL1 inhibition resulted in up-regulation of p53 protein in bronchial epithelial cells, then increased-p53 promoted autophagy. Autophagy in bronchial epithelial cells mediated collagen-I synthesis as well as airway remodeling in asthma.

**Conclusions:**

Taken together, our results suggest that BMAL1/p53-mediated bronchial epithelial cell autophagy contributes to PM2.5-aggravated asthma. This study highlights the functional importance of BMAL1-dependent p53 regulation during asthma, and provides a novel mechanistic insight into the therapeutic mechanisms of BMAL1.

**Graphic abstract:**

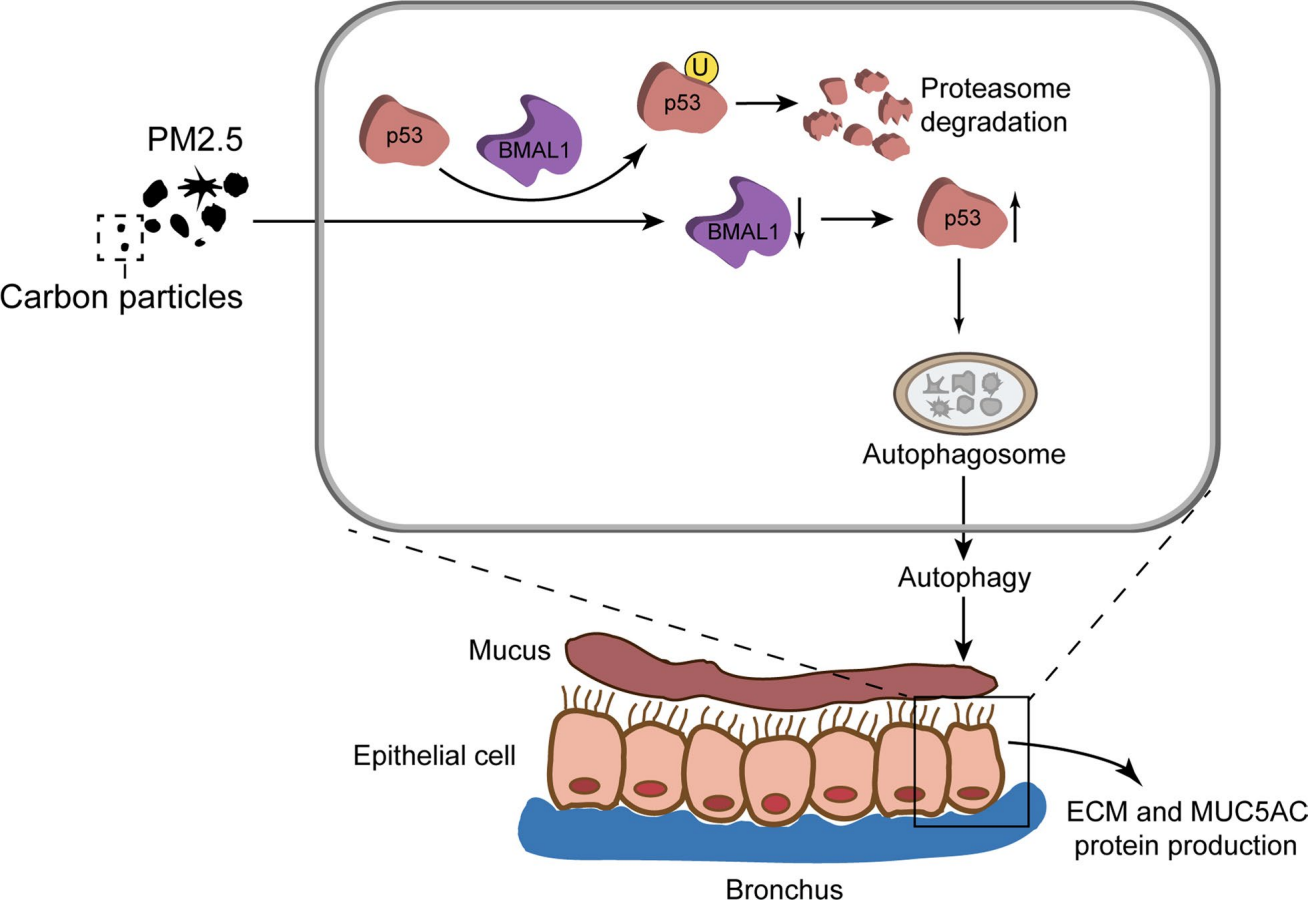

**Video Abstract**

**Supplementary Information:**

The online version contains supplementary material available at 10.1186/s12964-023-01057-9.

## Background

Owing to rapid industrialization, energy consumption, and population growth, many cities have been experiencing severe atmospheric particulate matter (PM) pollution [1–6]. PM2.5 is an atmospheric particulate matter with an aerodynamic diameter ≤ 2.5 μm. It has received global attention because of its harmful impact on health, especially airway diseases. Research supports the link between air pollution and an increased incidence and severity of airway diseases. Asthma is characterized by airway inflammation and bronchial hyper-responsiveness. The prevalence of asthma has increased remarkably across the globe, particularly in industrialized societies [7].

The airway epithelium lining the bronchial mucosa protects the respiratory mucosa through its physical barrier function, immune defense function, and mucociliary clearance of inhaled particles [8, 9]. Epithelial cell function is hampered by disruption of the epithelial barrier, and damage can facilitate the entry of pathogens, allergens, and pollutants into the body [10]. PM2.5 exposure disrupts tight junction proteins connecting airway epithelial cells, which could, in turn, instigate and sustain a PM-induced airway inflammatory response [11]. However, the detailed mechanisms underlying PM2.5-induced asthma and its exacerbation are still poorly understood.

The aryl hydrocarbon receptor nuclear translocator-like protein 1 (BMAL1) is a circadian clock transcriptional activator. BMAL1 is also extensively expressed in peripheral tissues and plays a crucial role in organs and tissue metabolism [12]. Zasłona et al.used mice lacking BMAL1 in myeloid cells to create an ovalbumin-induced asthma model and found dramatically increased asthmatic features, including increased lung inflammation, suggesting that the circadian protein BMAL1 is a negative regulator of asthma [13]. Ehlers et al. reported that deletion of BMAL1 in mice aggravated acute viral bronchiolitis caused by influenza A virus. BMAL1 knockout mice displayed heightened asthma-like airway changes after virus infection, including increased airway resistance and mucus production [14]. However, the role of BMAL1 in developing PM2.5-induced asthma is unknown.

Carbon particles are an important component of PM2.5. Janssen et al*.* proposed that carbon particles are an additional and valuable atmospheric quality indicator that could be used to assess the health risks of air pollution caused by primary combustion emissions and evaluate the benefits of traffic abatement measures [15]. In recent years, it has become increasingly evident that autophagy plays an important role in the development of asthma [16–18]. In this study, we used PM2.5 isolated from the atmosphere and carbon particles as particulate matter, we aimed to understand the role of BMAL1 and autophagy in asthma and PM2.5-challenged murine models and explore the underlying mechanisms.

## Results

### Carbon particles and PM2.5 aggravated airway remodeling in murine asthma models

**Fig. 1:**
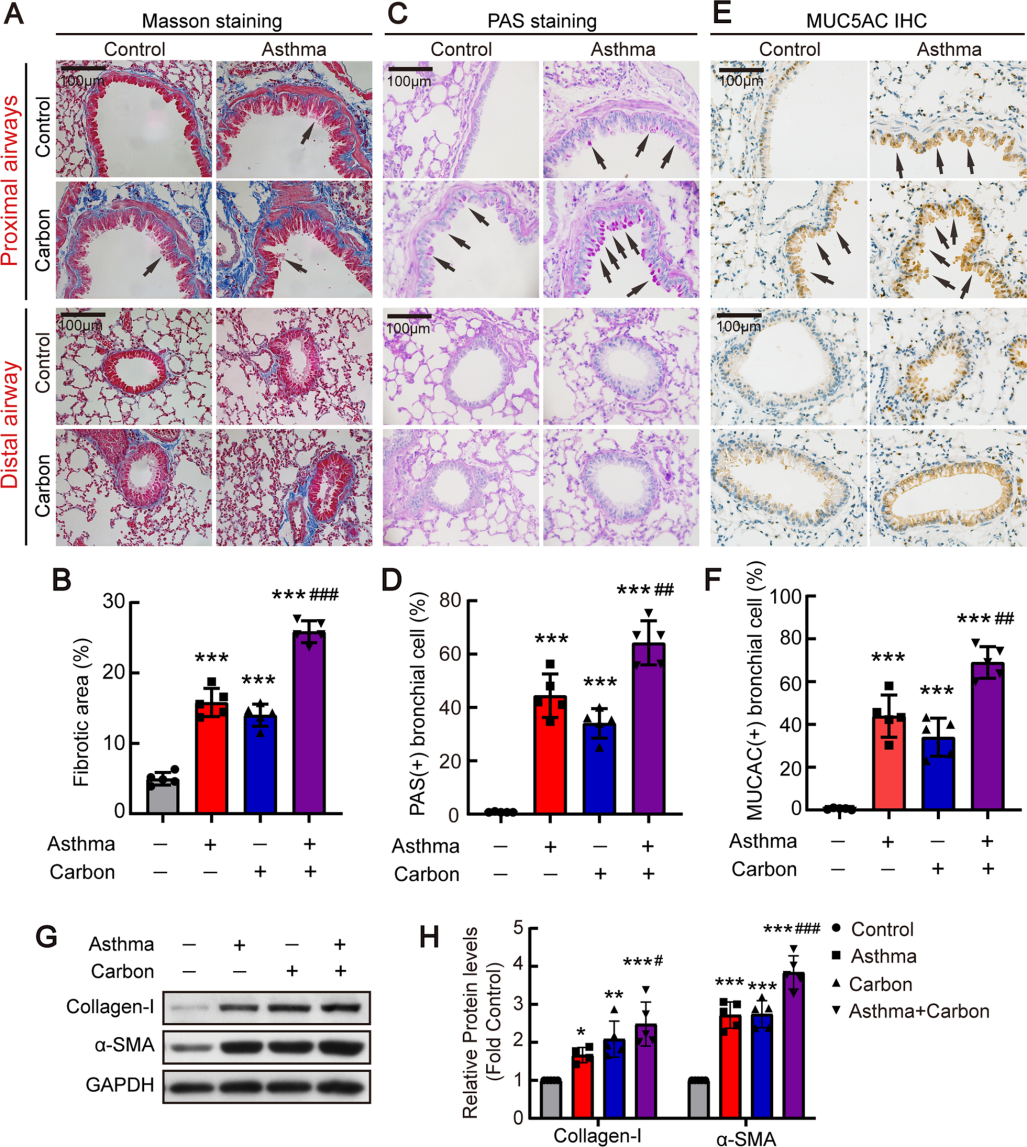
Carbon particles exposure aggravated airway remodeling in mouse chronic asthma model. Mice were sensitized with 10 mg OVA by intraperitoneal injection, and then administered by repeated aerosol challenges containing 3% OVA and carbon particles (2 mg/ml) over a 6-week period. The flow rate of ultrasonic atomized machine was 2 ml/min. Lungs of mice were harvested at day 60. **A** Representative images of proximal and distal intrapulmonary airways with Masson staining. Original magnification, ×400. **B** Collagen deposition area (%) of proximal airways in each group (n = 5). **C** Airway mucus secretion of proximal and distal intrapulmonary airways based on PAS staining (black arrows). Original magnification, ×400. **D** The quantification histograms of PAS staining in proximal airways (n = 5). **E** Representative images of immunohistochemistry staining of MUC5AC. Original magnification, ×400. **F** The quantification histograms of IHC MUC5AC staining in proximal airways (n = 5). **G** Representative immunoblots of collagen-I and α-SMA proteins in lung lysates.** H** Changes in protein levels of collagen-I (n = 5) and α-SMA (n = 6). Data are shown as mean ± SEM of n individual experiments. *P < 0.05, **P < 0.01, ***P < 0.001 (versus control); #*P* < 0.05, ##*P* < 0.01, ###*P* < 0.001 (versus asthma). *P* values were determined by one-way ANOVA followed by the Bonferroni’s test

To investigate effects of particulate matter on asthma, murine asthma models were generated by sensitization with ovalbumin (OVA). At the same time, some mice were exposed to particulate matters by administration of aerosols containing carbon particles formed by ultrasonic atomization. After 60 days, the mouse chronic asthma model was established. Murine lung tissues were stained using Masson’s Trichome to evaluate collagen deposition, and Periodic Acid-Schiff (PAS) and immunohistochemical (IHC) staining of mucin 5AC (MUC5AC) were performed to detect mucin secretion. As shown in Fig. 1A and B, airway epithelial hypertrophy and sub-epithelial fibrosis was evident in the proximal airway of asthmatic mice. Carbon particle exposure induced pathologic changes in mice similar to those seen in OVA-challenged mice. Furthermore, combined exposure to carbon particles and OVA aggravated sub-epithelial fibrosis compared with OVA-challenged alone. In the distal airway, epithelial denudation was observed in asthmatic mice and carbon particle-treated mice, without obvious sub-epithelial fibrosis. However, sub-epithelial fibrosis was found in mice treated with carbon particles plus OVA (Fig. 1A and B). As shown in Fig. 1C–F, PAS staining and IHC staining of MUC5AC revealed that OVA or exposure to carbon particles induced mucin secretion by goblet cells, which was enhanced by a combination of OVA and carbon particles. Moreover, mouse lung tissues were used to perform Western bloting. It revealed that collagen-I and alpha-smooth muscle actin (α-SMA) expression was increased in the lungs of mice treated with carbon particles or/and OVA (Fig. 1G and H). Similar to carbon particle exposure, PM2.5 exposure also augmented airway epithelial hypertrophy, sub-epithelial fibrosis, and mucin secretion in murine chronic asthma models (Additional file 1: Fig. S1).

To confirm the effect of particulate matter on asthma, mouse acute asthma models were also generated. An acute challenge was administered from days 21–27, and the manifestation of acute asthma was analyzed. As shown in Additional file 1: Fig. S2A, hematoxylin–eosin (HE) staining showed that carbon particles amplified asthmatic airway inflammation in both proximal and distal airways. Carbon particle exposure induced mucin secretion and MUC5AC expression (Additional file 1: Fig. S2B and C). Changes in mouse body weight are shown in Additional file 1: Fig. S2D. The number of cells in bronchoalveolar lavage fluid (BALF) was higher in asthmatic mice and carbon particle-treated mice than that in control. Additionally, carbon particles had an additive effect on the BALF cell number in asthmatic mice (Additional file 1: Fig. S2E and F). Protein levels of interleukin 4 (IL-4), interleukin 5 (IL-5), interleukin 6 (IL-6), interleukin 13 (IL-13), and MUC5AC in BALF were higher in the combined carbon particle-challenged asthmatic mice than those in asthmatic or carbon particle-challenged mice alone (Additional file 1: Fig. S2G–K). These data suggested that exposure to carbon particles deteriorated asthmatic airway inflammation and exacerbated asthma manifestation.

### BMAL1 was down-regulated in particulate matter-treated bronchial epithelial cells and particle matter-challenged mice

**Fig. 2:**
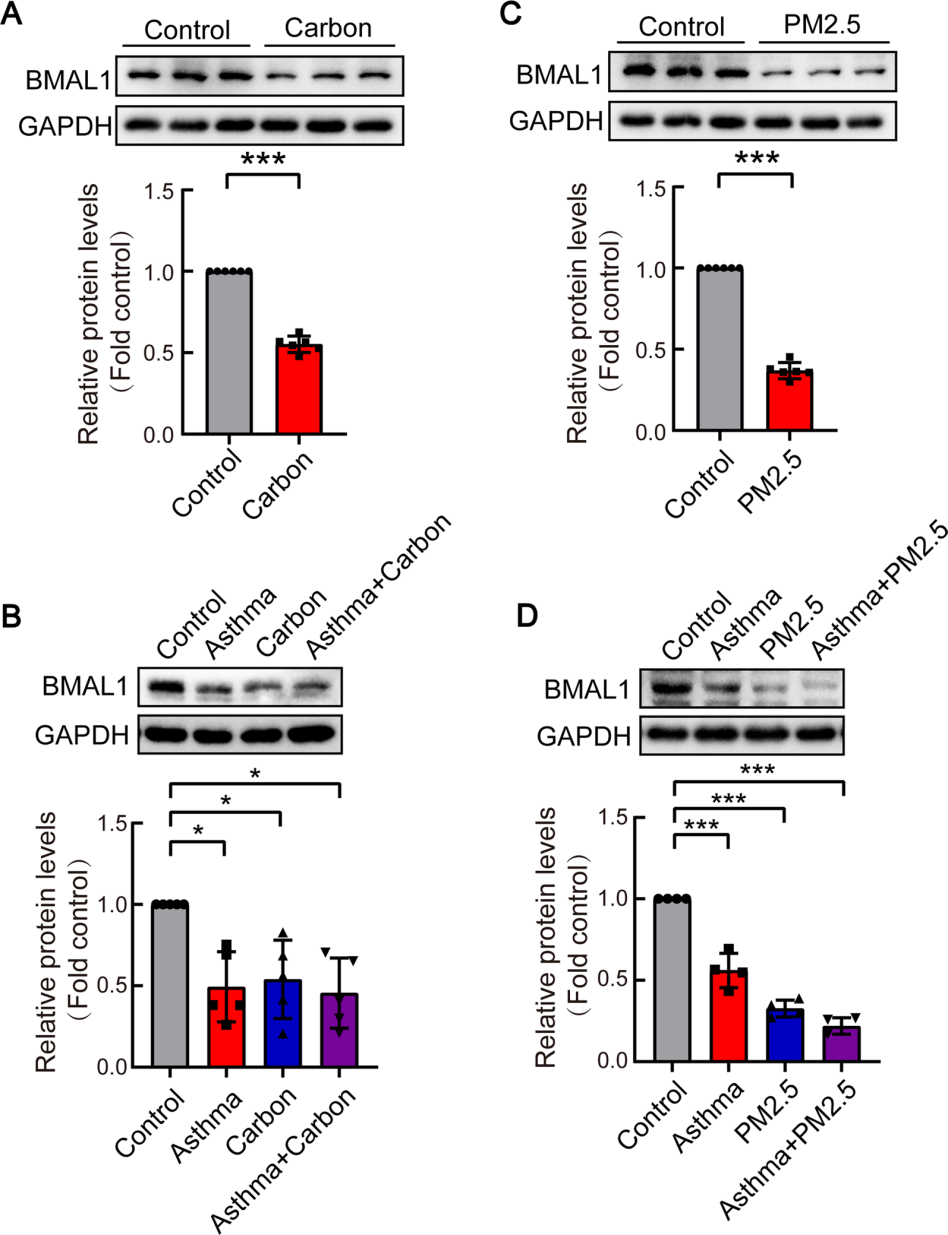
Carbon particles and PM2.5 induced BMAL1 down-regulation in vitro and in vivo. **A**, **C** HBE cells were treated with carbon particles (20 μg/ml) or PM2.5 (200 μg/ml) for 24 h. Whole-cell protein lysates were prepared for Western blot analysis using anti-BMAL1, n = 3. **B**, **D** In the mouse asthma model induced by OVA and carbon particles or PM2.5, BMAL1 protein contents were measured by western blotting, n = 5 (B), n = 4 (**D**). Data are mean ± SEM of n individual experiments. *P < 0.05, **P < 0.01, ***P < 0.001. *P* values were determined by Student’s *t* test (**A**, **C**) or one-way ANOVA followed by the Dunnett’s test (**B**, **D**)

As a circadian clock transcriptional activator, BMAL1 is also extensively expressed in peripheral tissues, and plays a crucial role in organs and tissue metabolism. To explore mechanisms by which particulate matter aggravates airway remodeling in asthma, BMAL1 protein levels were investigated. First, human bronchial epithelial cells (HBEs) were treated with carbon particles. As shown in Fig. 2A, BMAL1 protein levels were significantly lower in carbon particle-treated bronchial epithelial cells than that in control cells. BMAL1 protein levels were also reduced in lung tissues of asthma or/and carbon particle-challenged mice (Fig. 2B). In cells and mice exposed to PM2.5, changes of BMAL1 were similar to carbon particle treatment (Fig. 2C and D). Thus, both in vitro and in vivo results suggested that asthma and exposure to particulate matters reduced BMAL1 protein expression.

### BMAL1 mediated particulate matter-induced airway remodeling in bronchial epithelial cells

**Fig. 3:**
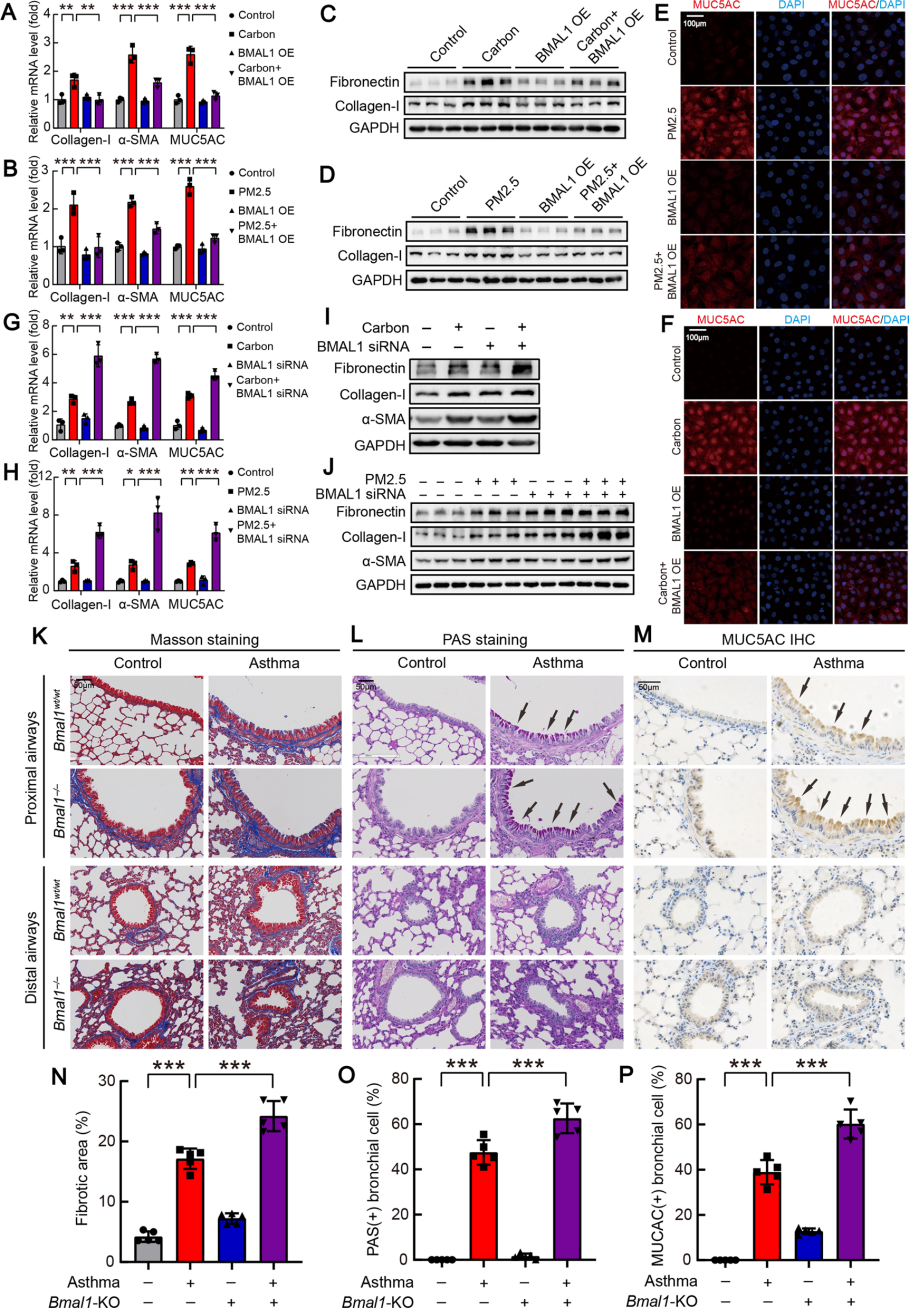
BMAL1 participated in carbon particles/PM2.5-induced airway remodeling in vitro and in vivo. **A**–**F** After transfection with control vector or BMAL1 expression vector for 72 h, HBEs were treated with carbon particles (20 μg/ml) or PM2.5 (200 μg/ml) for 24 h or 48 h. **A**, **B** mRNA changes of collagen-I, α-SMA and MUC5AC in HBEs. n = 3. **C**, **D** Representative blot images of collagen-I and fibronectin in HBEs. **E**, **F** Representative images of immunofluorescence staining of MUC5AC in HBEs stimulated by carbon particles/PM2.5. Scale bars: 100 μm. **G**–**J** After transfection with control or specific siRNA against BMAL1 for 48 h, HBEs were incubated with 20 μg/ml carbon particles or 200 μg/ml PM2.5, after which expression levels of related mRNA and protein were measured by the RT-qPCR (24 h) and western blotting (48 h). (G, H) mRNA changes of collagen-I, α-SMA and MUC5AC in HBEs. n = 3. **I**, **J** Representative blot images of collagen-I, fibronectin and α-SMA in HBEs. *Bmal1*^*−/−*^ mice and *Bmal1*^*wt/wt*^ mice were sensitized and challenged with OVA as described in the “Methods”. Lungs of mice were harvested at day 60. **K**–**M** Representative pictures of lung tissue sections stained with Masson staining, PAS staining and immunohistochemistry staining of MUC5AC. Scale bars: 50 μm. **N** Collagen deposition area (%) of proximal airways in each group (n = 5). **O** The quantification histograms of PAS staining in proximal airways (n = 5). **P** The quantification histograms of IHC MUC5AC staining in proximal airways (n = 5). Data are mean ± SEM of n individual experiments. *P < 0.05, **P < 0.01, ***P < 0.001 between indicated conditions (One-way ANOVA followed by the Bonferroni’s test)

To further explore the role of BMAL1 in particulate matter-induced airway remodeling, HBEs were treated with carbon particles or PM2.5. As shown in Additional file 1: Fig. S3A–F, carbon particles or PM2.5 up-regulated mRNA and protein levels of collagen-I, fibronectin, and α-SMA, as well as intracellular MUC5AC protein (Additional file 1: Fig. S3G). Next, BMAL1 overexpression was made in HBEs (Additional file 1: Fig. S4A). Carbon particles and PM2.5 induced increases of collagen-I, fibronectin, and MUC5AC proteins, but these were prevented by BMAL1-overexpression (Fig. 3A–F). Furthermore, BMAL1-specific siRNA was made (Additional file 1: Fig. S4B). BMAL1-siRNA, in addition to treatment with carbon particles or PM2.5, induced higher expression of fibronectin, collagen-I, α-SMA, and MUC5AC than carbon particles exposure alone (Fig. 3G–J). To further determine the role of BMAL1 in airway epithelial remodeling in vivo, we generated *Bmal1*^*−/−*^ mice and *Bmal1*^*wt/wt*^ mice (Additional file 1: Figs S4C and D). Staining of lung tissues revealed that asthma-challenged *Bmal1*^*−/−*^ mice displayed more severe bronchial epithelial cell hypertrophy, collagen deposition, and MUC5AC protein secretion compared with asthma-challenged *Bmal1*^*wt/wt*^ mice (Fig. 3K–P). Taken together, these data suggested that BMAL1 mediated particulate matter-induced airway remodeling in vitro and in vivo.

### BMAL1 regulated autophagy in bronchial epithelial cells

**Fig. 4:**
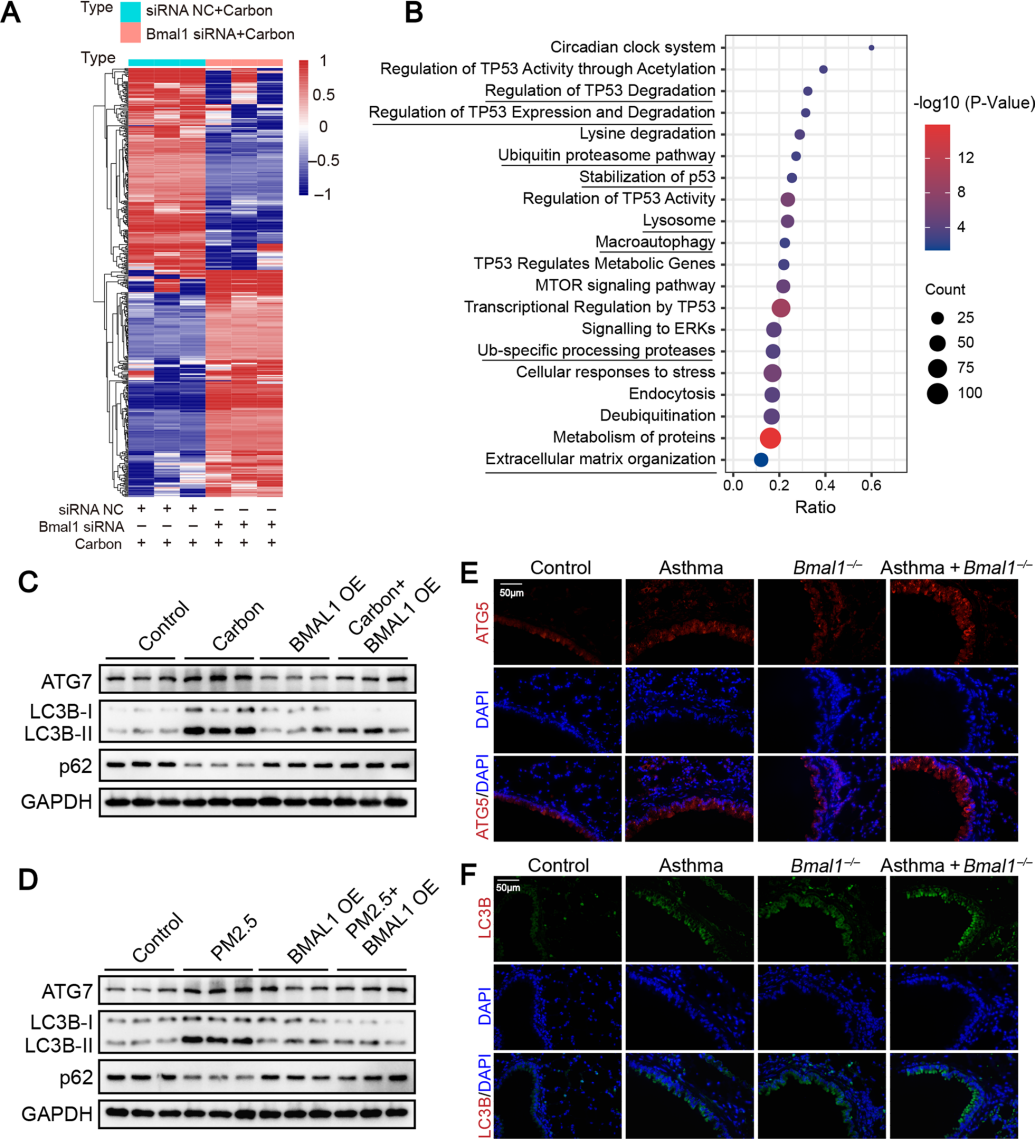
BMAL1 was involved in the process of autophagy induced by carbon particles/PM2.5 in vitro and in vivo. **A**, **B** mRNA sequencing of human HBEs after knockdown BMAL1 depletion. After transfection with human BMAL1 siRNA or negative control siRNA, HBEs were incubated with carbon particles (20 µg/ml) for 24 h, after which RNA sequencing was performed. **A** The heatmap of differentially expressed genes, FPKM (fragments per kilobase of transcript per million mapped reads) values were represented by gradient colors and shown for each sample. Red represented a higher FPKM; blue represented a lower FPKM. Results were based on 3 RNA sequencing samples. **B** Pathway enrichment analysis showed differentially expressed genes that related to different pathways terms. **C**, **D** After transfection with control vector or BMAL1 overexpression vector (BMAL1 OE) for 72 h, HBEs were treated with carbon particles (20 μg/ml) or PM2.5 (200 μg/ml) for 48 h. Representative blot images of ATG7, LC3B-II and p62 in HBEs. **E**, **F** *Bmal1*^*−/−*^ mice and *Bmal1*^*wt/wt*^ mice were sensitized and challenged with OVA and carbon particles as described in the Methods. Lungs of mice were harvested at day 60. Representative pictures of lung tissue sections stained with ATG5 and LC3B. Scale bars: 50 μm

To understand how BMAL1 regulates airway epithelial remodeling induced by carbon particles, RNAs from HBEs treated with carbon particles or/and BMAL1 siRNA were sequenced. As shown in Fig. 4A and Additional file 1: Fig. S5A, there were 486 differentially expressed genes in the cells treated with BMAL1-siRNA compared with negative control siRNA (siRNA NC), out of which 229 genes were up-regulated, and 257 genes were down-regulated. The differentially expressed genes were involved in diverse pathways, including the circadian clock system, regulation of p53, autophagy, and extracellular matrix organization (Fig. 4B). Based on gene ontology (GO) enrichment analysis, these genes were found mainly enriched in autophagy and regulatory of p53 (Additional file 1: Fig. S5B). Most of these analyses indicated that BMAL1 was closely related to autophagy, regulation of p53, and extracellular matrix remodeling. The important role of BMAL1 in autophagy has been previously documented [19]. However, relationships among BMAL1, autophagy and particulate matter were poorly understood in bronchial epithelial cells.

Then, after treatment with particles matters, autophagosomes, autophagy-related markers were detected in bronchial epithelial cells. Carbon particles or PM2.5 induced production of autophagosomes (Additional file 1: Fig. S6A), increases of LC3B-II, collagen-I, fibronectin, α-SMA and autophagy-related 5 (ATG5), decreases of sequestosome 1 (p62) (Additional file 1: Fig. S6B and C). Moreover, carbon particles or PM2.5 also increased ATG5 and LC3B expression in mouse models (Additional file 1: Fig. S6D and E). On the contrary, upregulation of autophagy-related proteins induced by particulate matter was reversed by BMAL1-overexpressing in the cells (Fig. 4C and D). Next, *Bmal1*^*−/−*^ mice and *Bmal1*^*wt/wt*^ mice were used to reveal autophagy levels in vivo. The asthma-challenged *Bmal1*^*−/−*^ mice had higher autophagy marker expression than that in asthma-challenged *Bma1l*^*wt/wt*^ mice (Fig. 4E and F).

Next, the role of autophagy induced by particulate matter in airway epithelial remodeling was investigated. The autophagy inhibitor hydroxychloroquine (HCQ) and ATG5-siRNA was used to inhibit autophagy in HBEs. HCQ inhibits autophagosome-lysosome fusion, so HCQ inhibits autophagy but increases LC3B-II levels. First, high LC3B-II levels were confirmed in the HBEs (Additional file 1: Fig. S7A-C). HCQ treatment reversed carbon particle-induced up-regulation of collagen-I, fibronectin, ATG5, α-SMA, and MUC5AC, and down-regulation of p62 (Additional file 1: Fig. S7A, B and D). HCQ also reversed PM2.5 induced up-regulation of collagen-I, fibronectin, and α-SMA (Additional file 1: Fig. S7E and F). More importantly, we also attenuated autophagy by knockdown of the essential autophagy proteins ATG5 using siRNA. ATG5-siRNA depressed LC3B-II expression, and similar to HCQ, ATG5-siRNA reversed carbon particle/PM2.5 induced up-regulation of collagen-I, fibronectin, and α-SMA (Additional file 1: Fig. S8). These results suggested that BMAL1 regulated autophagy in bronchial epithelial cells, and autophagy is involved in airway epithelial remodeling which induced by particulate matter.

### BMAL1 regulated particulate matter-induced autophagy via *p53*

**Fig. 5:**
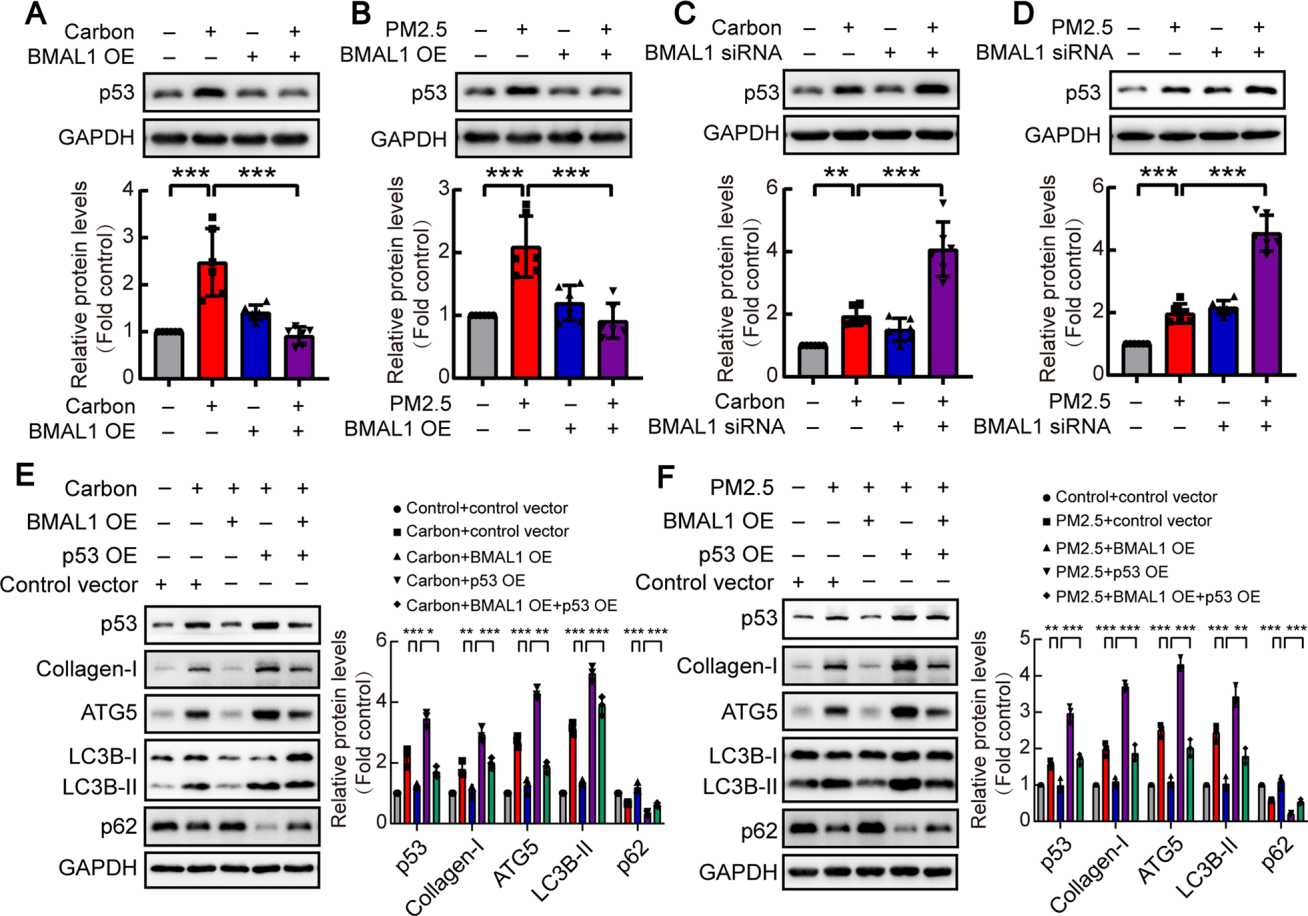
BMAL1 regulated carbon particles/PM2.5-induced autophagy via p53 protein. HBEs were incubated with control vector or BMAL1 expression vector for 72 h and treated with PM2.5 (200 μg/ml) or carbon particles (20 μg/ml) for 24 h prior to harvest of cell lysates, after which expression levels of p53 protein were measured by western blotting. **A**, **B** Representative images of Western blots of p53 were shown, and changes in relative density of p53 were presented. n = 6. **C**, **D** After transfection with human BMAL1 siRNA or negative control siRNA, HBEs were incubated with carbon particles or PM2.5 for 24 h, after which p53 protein were measured by western blotting and changes in relative density of p53 were presented. n = 6. **E**, **F** HBEs were incubated with control vector, BMAL1 expression vector or p53 expression vector for 72 h and treated with PM2.5 (200 μg/ml) or carbon particles (20 μg/ml) for 48 h prior to harvest of cell lysates, after which expression levels of p53, collagen-I, ATG5, LC3B-II, and p62 protein were measured by western blotting. Representative images of Western blots were shown, and changes in relative density of protein were presented. n = 3. Data are mean ± SEM of n individual experiments. *P < 0.05, **P < 0.01, ***P < 0.001 between indicated conditions (One-way ANOVA followed by the Bonferroni’s test)

Based on the RNA sequencing results presented above, p53-related signaling pathways and autophagy were significantly enriched in the pathway analysis. Since BMAL1 has been reported to regulate p53 transcriptional activity [20, 21], we speculated that BMAL1 may regulate p53 protein levels in HBEs treated with carbon particles. To test this hypothesis, HBEs were treated by BMAL1-siRNA along with carbon particles or PM2.5, and then p53 protein expression was examined. Carbon particle/PM2.5 exposure up-regulated p53 expression, and these changes were prevented by BMAL1-overexpressing in the cells (Fig. 5A and 5B). On the contrary, BMAL1-siRNA augmented the up-regulation of p53 in carbon particle/PM2.5-treated cells (Fig. 5C and D). These observations suggested that BMAL1 regulates p53 in bronchial epithelial cells stimulated by particulate matter. Furthermore, p53 plays a key role in regulating autophagy [22–24]. We then studied whether BMAL1 mediates cell autophagy via p53 in bronchial epithelial cells. As shown in Fig. 5E and F, BMAL1 overexpression attenuated p53 expression as well as cell autophagy. The p53 overexpression augmented autophagy, but BMAL1 overexpression relieved autophagy which regulated by p53 in the cells. These results suggested that BMAL1/p53 pathway regulated carbon particle/PM2.5-induced autophagy in airway epithelial cells.

### BMAL1 directly bound p53 protein via structural domains and regulated its stability

**Fig. 6:**
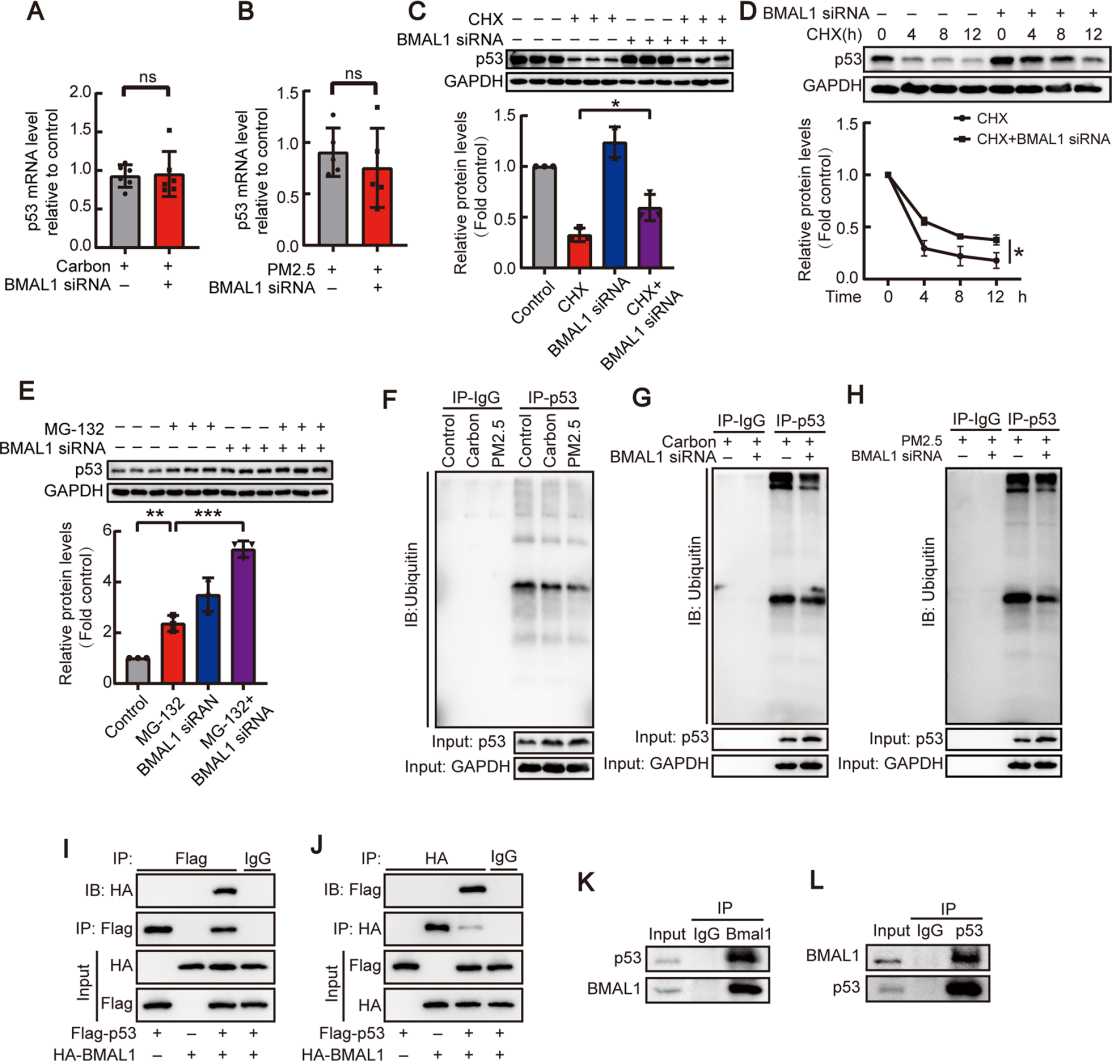
BMAL1 directly bound to p53 and regulated its protein stability. **A**, **B** HBEs were incubated for 48 h after transfection with control siRNA or specific siRNA against BMAL1 and treated with carbon particles (20 μg/ml) or PM2.5 (200 μg/ml) for 24 h, after which p53 mRNA levels were measured by RT-qPCR. (A) n = 6. **B** n = 5. *P < 0.05 versus the control group (Student’s *t*-test). **C**, **D** After transfection with control or specific siRNA against BMAL1 for 48 h, HBEs were incubated with CHX for 4 h or timing gradients, after which levels of p53 protein were measured by western blot analysis. Changes in relative density of p53 to GAPDH were presented. **E** After transfection with control or specific siRNA against BMAL1 for 48 h, HBEs were incubated with MG-132 for 2 h, after which the levels of p53 protein were measured. Changes in relative blot density of p53 to GAPDH were presented. **F**–**H** HBEs were treated with carbon particles or PM2.5; or transfected with control or specific siRNA against BMAL1 and carbon particles or PM2.5 for 24 h. Immunoprecipitation assay was accomplished with anti-p53 or anti-IgG antibody and the endogenously precipitated complexes were detected with anti-ubiquitin antibody. **I**, **J** HEK293T cells were cotransfected with Flag-p53 and control vector; or HA-BMAL1 and control vector; or Flag-p53 and HA-BMAL1 expression vector for 72 h respectively. The co-immunoprecipitation assay was performed with anti-Flag, anti-HA or anti-IgG antibody and the precipitated complexes were detected with anti-HA or anti-Flag antibodies. **K**, **L** HBEs were used for the immunoprecipitation experiments. The experiments were carried out with anti-p53, anti-BMAL1 or anti-IgG antibody and the endogenously precipitated complexes were detected with anti-p53 or anti-BMAL1 antibody. Data are mean ± SEM of n individual experiments, n = 3. *P < 0.05, **P < 0.01, ***P < 0.001 between indicated conditions (One-way ANOVA followed by the Bonferroni’s test)

To investigate the detailed mechanism by which BMAL1 regulated p53, p53 mRNA and protein levels were studied. HBEs were treated with BMAL1 siRNA followed by carbon particles or PM2.5, and then p53 mRNA and protein levels were analyzed. As shown in Fig. 6A and B, there was no significant change in p53 mRNA levels in cells treated with BMAL1-siRNA, indicating that BMAL1 did not affect p53 mRNA expression. Based on RNA sequence analysis, the differentially expressed genes were enriched in pathways involved in the regulation of p53 degradation, stabilization of p53, and the ubiquitin–proteasome pathway. Thus, the role of BMAL1 in regulating p53 protein levels was investigated. Accordingly, cycloheximide (CHX) was used to inhibit protein synthesis in HBE cells treated with BMAL1-siRNA. After CHX inhibited protein synthesis, BMAL1-siRNA treated HBE cells displayed higher p53 protein levels than those in control HBE cells (Fig. 6C). Meanwhile, BMAL1-siRNA attenuated the rate of p53 protein degradation (Fig. 6D). To further explore the role of BMAL1 in regulating p53, a proteasome inhibitor (MG132) was added to BMAL1-siRNA transfected HBEs. As shown in Fig. 6E, MG132 increased p53 protein levels, and BMAL1-siRNA aggravated the effect. This indicated that BMAL1 repressed p53 protein levels probably via the ubiquitin/proteasome pathway. Next, co-immunoprecipitation (Co-IP) was used to examine whether BMAL1 binds p53 and promotes its ubiquitination. As shown in Fig. 6F, levels of ubiquitinated p53 were significantly reduced in carbon particles or PM2.5 treated HBEs. Depleting BMAL1 with BMAL1-siRNA further decreased the ubiquitinated p53 in carbon-particle or PM2.5 treated HBEs (Fig. 6G and H). This observation confirmed that BMAL1 regulated p53 ubiquitination. Co-immunoprecipitation was also used to verify the interaction between BMAL1 and p53 and showed that BMAL1 bound p53 (Fig. 6I–L).

**Fig. 7:**
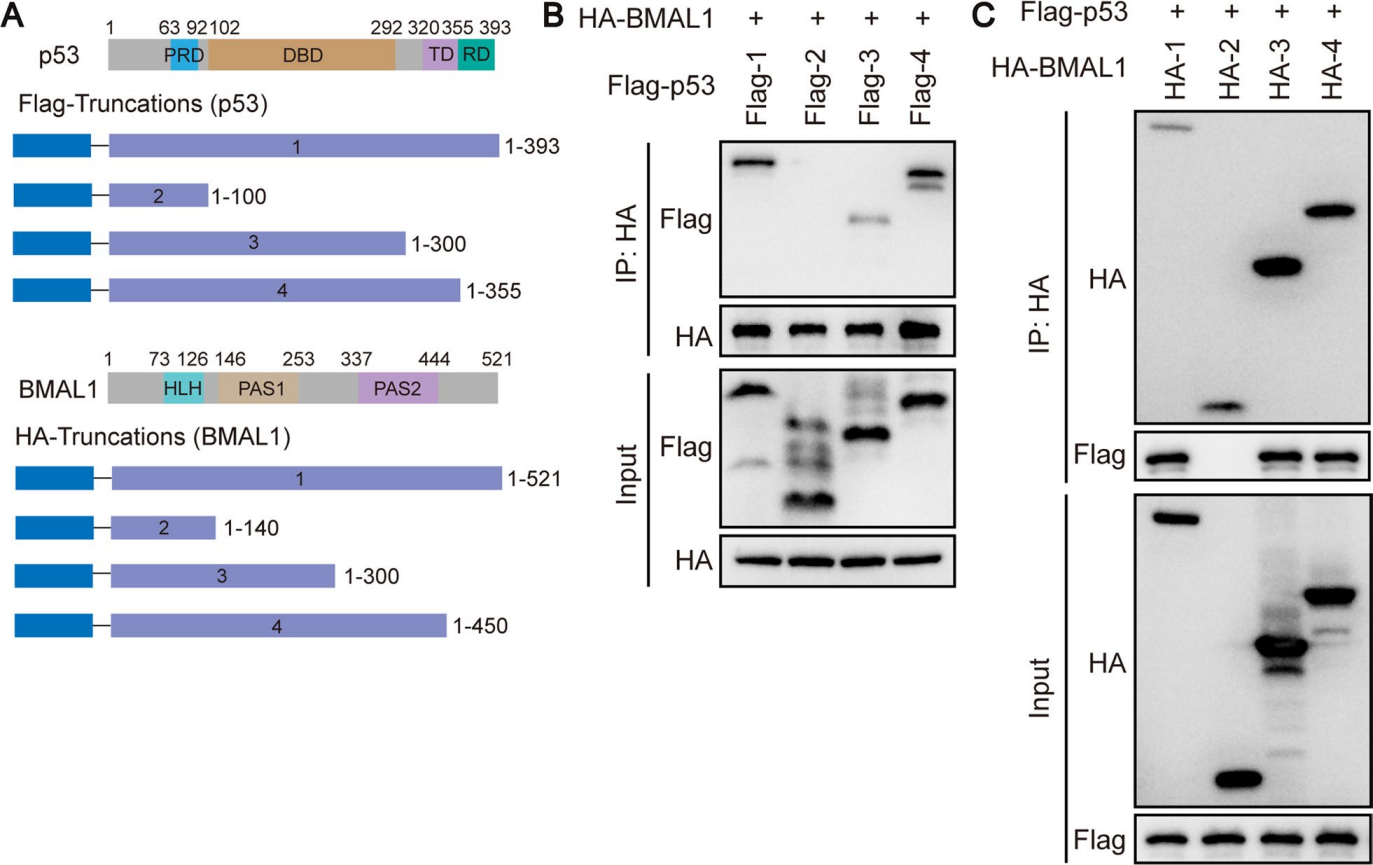
BMAL1 interacted with p53 via particular structural domains. **A** The strategy for Flag-p53 and HA-BMAL1 truncations. Different structural domains were represented by different color blocks, the specific details have been marked in the Fig. PRD, proline-rich domain; DBD, DNA binding domain; TD, tetramerization domain; RD, regulatory domain; HLH, helix-loop-helix domain; PAS: PER-ARNT-SIM domain. **B**, **C** Determination of p53 and BMAL1 domains required for their interaction with each other. Different p53-truncated constructs were co-transfected with HA-tagged BMAL1 and different BMAL1-truncated constructs were co-transfected with Flag-tagged p53 into HEK293T cells. The cells were then subjected to co-immunoprecipitation and Western blot analyses with the indicated antibodies

To determine the structural basis for interaction between protein BMAL1 and p53, we constructed a series of truncated fragments fused with different tags that included FLAG and HA (Fig. 7A). We observed that the DNA binding domain of p53 directly bound PER-ARNT-SIM domain (PAS1) of BMAL1 (Fig. 7B and C).

Collectively, the above results suggested that PAS1 domain of BMAL1 directly bound DNA binding domain of p53, regulated p53 ubiquitination, and thus promoted p53 degeneration. Conversely, when particulate matters decreased BMAL1 levels, low level BMAL1 resulted to high stabilization of p53, as well as increased p53 protein levels.

### p53 knockdown reversed particulate matter-mediated increase in collagen-I and MUC5AC levels in bronchial epithelial cells

**Fig. 8:**
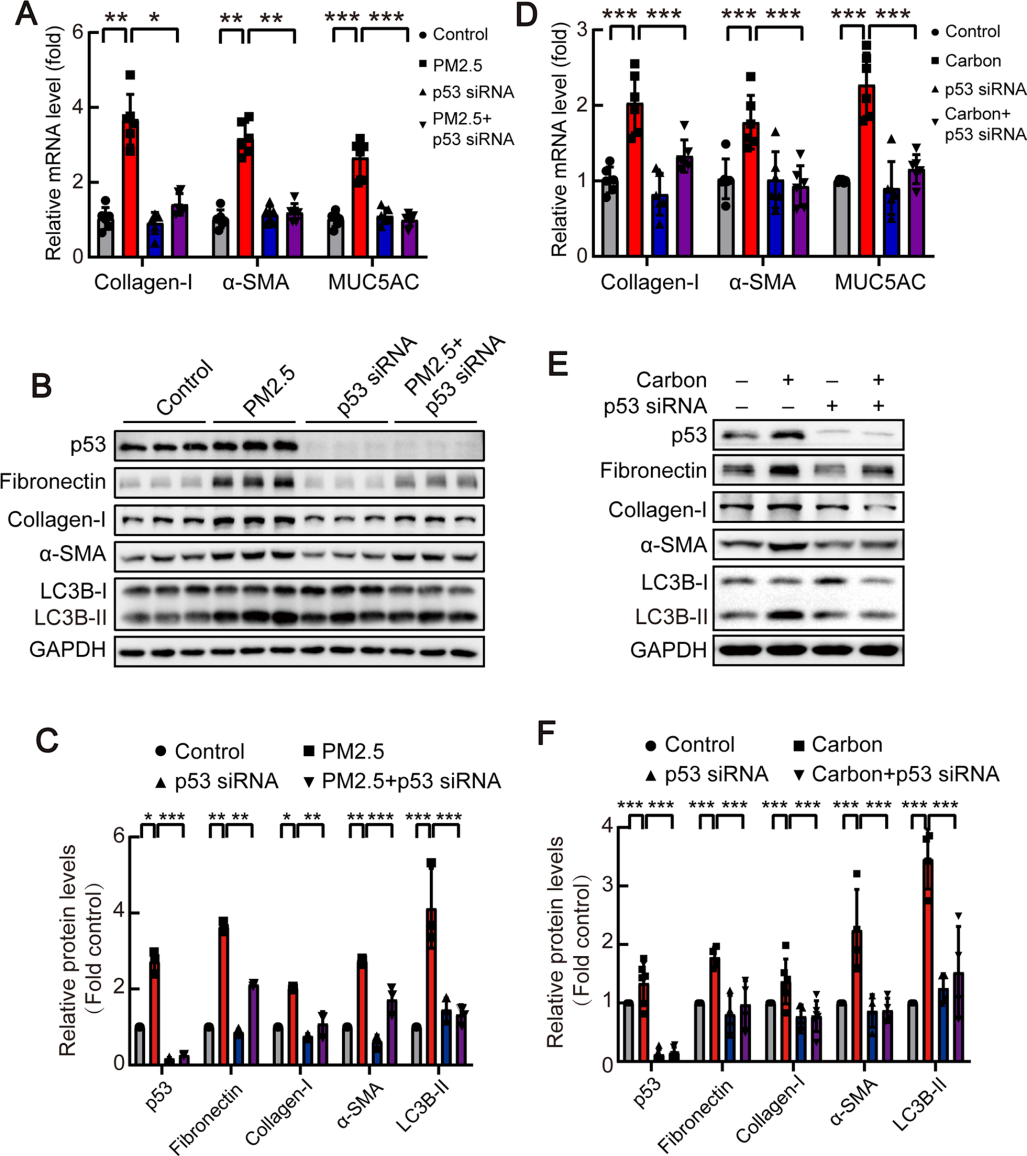
p53 siRNA prevented PM2.5/carbon particle-induced collagen-I expression and cell autophagy in bronchial epithelial cells*.* HBEs were incubated for 48 h after transfection with control or specific siRNA against p53 and treated with carbon particles (20 μg/ml) or PM2.5 (200 μg/ml) for 48 h prior to harvest of cell lysates, after which expression levels of protein were measured by qRT-PCR and Western blots. **A**, **B** mRNA changes of collagen-I, α-SMA and MUC5AC in HBEs. n = 6. **C**, **E** Representative images of Western blots of p53, fibronectin, collagen-I, α-SMA and LC3B-II were shown. **D**, **F** The changes in relative density of p53, fibronectin, collagen-I, α-SMA and LC3B-II to GAPDH were presented. n = 3 (**C**); n = 6 (p53 and collagen-I in **D**), n = 4 (fibronectin, α-SMA and LC3B-II in **D**). Data are mean ± SEM of n individual experiments. *P < 0.05, **P < 0.01, ***P < 0.001 between indicated conditions (One-way ANOVA followed by the Bonferroni’s test)

To further validate the role of p53 in airway remodeling, p53-siRNA was used to knock down p53 in bronchial epithelial cells. As shown in Fig. 8A, p53-siRNA blocked PM2.5-induced up-regulation collagen-I, α-SMA, and MUC5AC mRNAs in bronchial epithelial cells. PM2.5-induced the autophagy marker LC3B-II and increased remodeling proteins fibronectin and collagen-I, but autophagy and airway remodeling were prevented by p53-siRNA in bronchial epithelial cells (Fig. 8B and C). In cells exposed to carbon particles (Fig. 8D-F), p53-knockdown induced similar changes. These data indicated that p53 was exactly involved in airway epithelial remodeling and MUC5AC expression.

## Discussion

In this study, we confirmed that PM2.5 aggravated airway remodeling in murine chronic asthma and exacerbated asthma manifestation in murine acute asthma. Next, low BMAL1 expression was found to be crucial for airway remodeling in PM2.5-challenged asthmatic mice. Subsequently, we showed that BMAL1 could bind and promote the ubiquitination of p53, which can regulate p53 degradation and block its increase under normal conditions. However, PM2.5-induced BMAL1 inhibition resulted in up-regulation of p53 protein in bronchial epithelial cells, then increased-p53 promoted autophagy. Autophagy in bronchial epithelial cells mediated collagen-I synthesis and MUC5AC expression as well as airway remodeling in asthma (Graphical Abstract).

The atmospheric concentration of PM2.5 positively correlates with an increase in the risk of hospitalization for respiratory diseases, including asthma [25]. Fan J et al*.* reported that PM2.5 had an adverse impact on emergency department visits for asthma when PM2.5 concentration was high [26]. Zhao C and colleagues found that soluble PM2.5 extract contributed to airway barrier dysfunction. They hypothesized that in response to PM2.5, airway epithelial cells produce soluble mediators, which orchestrate the breaking of inhalational tolerance and sensitization to allergic antigens, as well as exacerbate the development of asthma [27]. Thus, several studies support the notion that PM2.5 aggravates asthma. Consistent with these studies, we found that PM2.5 and carbon particles aggravated airway remodeling in murine chronic asthma and exacerbate asthma manifestation in murine acute asthma models.

PM2.5 can induce tissue remodeling, including cardiovascular, nasal, and airway remodeling. In our study, we confirmed that PM2.5 and carbon particles induced remodeling in the airway and enhanced the synthesis of the classical remodeling protein collagen-I in bronchial epithelial cells. Yue W et al*.* reported that PM2.5 exposure caused vascular remodeling and instigated a shift from left ventricular failure to right ventricular hypertrophy [28]. A high concentration of PM2.5 in the atmosphere was significantly correlated to the remodeling of the coronary artery [29]. Nasal remodeling and mucus hypersecretion in an allergic rhinitis rat model was also found after PM2.5 treatment [30]. PM2.5 increased some epidermal growth factor receptor ligands in airway epithelial cells, which elicited and sustained a pro-inflammatory response and contributed to airway remodeling [31]. Dysart MM et al*.* found that PM2.5 augmented the activation of TGF-β in stiffness-challenged alveolar epithelial cells, which contributed to lung remodeling during fibrosis [32]. PM2.5 promoted the migration of human bronchial smooth muscle cells via the sonic hedgehog pathway, which mediated PM2.5-induced airway remodeling in chronic obstructive pulmonary disease (COPD) [33]. Moreover, researchers recently discovered that PM2.5 induced changes in T cells and macrophages, which were involved in tissue remodeling. Analysis from Guo ZQ and colleagues revealed an increase in the immune response with Th2 polarization and the development of nasal remodeling-mediated exacerbation of allergic rhinitis after exposure to PM2.5 [34]. PM2.5 facilitated M2 macrophage polarization, thus, promoting airway remodeling during the development of PM2.5-induced COPD [35]. Therefore, PM2.5-induced tissue remodeling exacerbates diseases, especially respiratory disorders.

Low BMAL1 expression was involved in airway remodeling in PM2.5-challenged asthmatic mice in the current study. p53 is a tumor suppressor, and studies regarding pancreatic cancer have shown that BMAL1 promotes p53 transcription by directly binding to the p53 promoter, thereby transcriptionally activating the downstream tumor suppressor pathway in a p53-dependent manner [20]. In our study, while the knockdown of BMAL1 did not change the p53 mRNA levels in airway epithelial cells, it affected p53 protein stability by directly binding to it. Therefore, deficiency of BMAL1 reduces the proteasomal degradation of p53 and promotes the accumulation of p53 protein. It seems that BMAL1 protein regulates p53 differently in different diseases. It has been reported that BMAL1 binds to gene promoter regions with high tissue specificity, showing only a slight overlap between different organs [36, 37]. BMAL1 regulates its downstream targets not only through transcriptional activation or repression but it also participates in the post-translational regulation of proteins [38, 39]. Therefore, we thought that the mechanism of BMAL1-mediated p53 protein regulation may be tissue-specific and form diversity. Our study may propose a novel role for BMAL1 in regulating p53 stability in airway epithelial cells.

We also investigated p53-related mechanisms in airway remodeling during asthma. Several studies have highlighted the role of autophagy in airway remodeling in asthma. McAlinden KD and colleagues found that selective activation of autophagy occurs in the structural cells during asthma [18]. Another study suggested that activation of autophagy-mediated epithelial-mesenchymal transition contributed to airway remodeling [40]. MicroRNA such as microRNA-30a target ATG5 and attenuate airway fibrosis in asthma by inhibiting autophagy [41]. The autophagy inhibitor chloroquine showed effective preclinical efficacy in airway remodeling of murine asthma models [18]. Exposure to PM2.5 can induce autophagy in various cells, including bronchial epithelial cells, alveolar epithelial cells, and vascular endothelial cells [42–44]. RNA sequencing analysis was performed on BMAL1 knockdown airway epithelial cells stimulated with carbon particles. The differentially expressed genes were mainly enriched in pathways involved in autophagy and p53 regulation. We found that PM2.5 and carbon particles induced bronchial epithelial cell autophagy, and airway epithelial remodeling which was relieved in the presence of autophagy inhibitors. p53 regulates autophagy through a variety of interactions and signaling pathways. p53 has two target genes, *Sestrin1* and *Sestrin2*, which activate the AMP-responsive protein kinase as well as induce it to phosphorylate TSC2 and stimulate its GTPase activating protein activity, thereby inhibiting the mammalian target of rapamycin [45]. p53 promotes PTEN-mediated autophagy as it suppresses TORC1 activity [46]. p53 also can dephosphorylate extracellular signal-regulated kinase 1/2 (ERK1/2), thereby inhibiting ERK1/2 signaling [47]. Lee HJ reported that ERK1/2 is also an upstream target of p53 during apoptosis of aortic endothelial cells [48]. There may be crosstalk between multiple signaling pathways in the process of p53-induced autophagy. Thus, the regulation of p53-induced autophagy is complicated, and future studies are required to explore the specific signaling pathways underlying p53-induced autophagy in asthma.

In conclusion, we showed that BMAL1 regulated autophagy via p53 in airway epithelial cells, which contributed to PM2.5-aggravated asthma.

## Methods

### Materials and reagents

Carbon particles with diameters of 24 nm were purchased from Mitsubishi Chem5-ical Corporation (Tokyo, Japan, MA-100). Ovalbumin (OVA) was purchased from Sigma-Aldrich (St. Louis, MO, USA, A5253). Aluminium hydroxide was purchased from Sangon Biotech (Shanghai, China). Rapamycin (S1039), Torin1 (S2811), cycloheximide (CHX, S7418) and MG-132 (S2619) were purchased from Selleck (Selleck Chemicals, Shanghai, China). Hydroxychloroquine (HCQ, HY-17589A) was obtained from MCE (Shanghai, China). LY3214996 was obtained from Topscience Technology Limited Company (Shanghai, China). The tandem GFP‐RFP‐LC3 adenovirus construct was purchased from Jikai Co. (Shanghai, China). Anti-BMAL1 (ab3350) antibody was abtained from Abcam (Cambridge, UK). Anti-phospho-mTOR (#5536), anti-mTOR (#2972), anti-phospho-ERK1/2 (#4370S), anti-ERK1/2 (#4695S), anti-phospho-AKT (#4060S) and anti-AKT (#9272S) were purchased from Cell Signaling Technology (Danvers, MA, USA). Anti-α-SMA (14,395–1-AP), anti-fibronectin (15,613–1-AP), anti-LC3B (14,600–1-AP), anti-ATG5 (10,181–2-AP), anti-p62 (18,420–1-AP), anti-p53 (10,442–1-AP), anti-BHLHE40 (17,895–1-AP), anti-TGF-β1 (21,898–1-AP) and anti-GAPDH (60,004–1) were purchased from Proteintech Technology (Wuhan, China). Anti-MUC5AC was obtained from absin (Shanghai, China). Anti-collagen-I was purchased from ABZOOM (Shanghai, China).

### PM2.5 sample preparation

PM2.5 was collected between November 2019 and March 2020 in Wuhan using a large-flow PM2.5 sampler (MSP, USA, UAS-310). The sampling site was located on the roof of a five-story building on the campus of Tongji Medical College. The PM2.5 samples were collected and prepared according to the following procedure. With an air flow rate of 1.05 m^3^/min, the PTFE microfiltration membrane (Safelab, China, Beijing) was utilized to collect PM2.5. The microfiltration membrane was subsequently presoaked in sterile water for 30 min. PM2.5 were extracted from the membrane via sonication, centrifugation and vacuum freeze drying. The dried PM2.5 was weighed and stored at − 20 °C until use.

### Cell culture and transfection

Human normal bronchial epithelial cell line HBE (CRL-2741) and human embryonic kidney HEK293T (CRL-1573.3) were obtained from American Type Culture Collection and cultured in high-glucose DMEM medium (Gibco, Grand Island, NY, USA, C11995500BI) supplemented with 10% fetal bovine serum (Gibco, 10,100,147) and 10% newborn calf serum (Gibco, A3520502), respectively. Penicillin (100 U/ml) and streptomycin (100 μg/ml) were added to all media. HBEs and HEK293T were maintained in a humidified atmosphere at 37 °C containing 5% CO2. For overexpression experiments, HBEs and HEK293T were transfected with Neofect™ (Neofect Biotechnologies, Shanghai, China, TF201201) according to the instruction manual. Transfected HBEs and HEK293T were harvested 72 h after treatment. For siRNA transfection, Lipofectamine RNAiMAX reagents (Invitrogen, Carlsbad, USA, 2,297,560) and specific gene siRNA (IBSBIO, Shanghai, China) were added into serum-free medium according to the manufacturer’s protocol. The sequences for siRNAs targeting specific gene were as follow: BMAL1 siRNA 5’-UAG GCA CAU CGU GUU AUG AAU TT-3’, ATG5 siRNA 5’- GAA GGU UAU GAG ACA AGA AGA-3’, p53 siRNA 5’-CUA CUU CCU GAA AAC AAC GUU-3’.

### Plasmids

The pcDNA3.1-p53-Flag plasmid and pcDNA3.1-BMAL1-HA plasmid were purchased from Genomeditech (Shanghai, China). The pcDNA3.1-p53-Flag-2 (1-100 aa), pcDNA3.1-p53-Flag-3 (1-300 aa), pcDNA3.1-p53-Flag-4 (1-355 aa), pcDNA3.1-BMAL1-HA-2 (1-140 aa), pcDNA3.1-BMAL1-HA-3 (1-300 aa), and pcDNA3.1-BMAL1-HA-4 (1-450 aa) plasmids were purchased from Genecreate (Wuhan, China). All vectors were confirmed by DNA sequencing.

### Western blot

Mice lung tissues or HBEs cells were homogenized with ice-cold lysis buffer containing a phosphatase inhibitor (Roche, Basel, Switzerland, 539,142) and protease inhibitor cocktail (Calbiochem, San Diego, CA, 539,131), and the protein concentration of the samples was estimated with BCA assay kit (Thermo, Waltham, MA, 23,225). Western blot was conducted according to the standard protocol. Protein extracts were loaded into 7.5–12.5% SDS polyacrylamide gels (120 V, 90 min). The proteins were transferred to PVDF membranes (Roche, 49,916,800) (280 mA, 90 min), and the PVDF membranes were incubated with primary antibody at 4 °C overnight. The next day, the membranes were incubated with the appropriate HRP-conjugated secondary antibody for 1 h, followed by HRP activity-based signal detection. Band intensity was measured, when indicated, using IpWin5 software.

### RNA extraction and quantitative real-time polymerase chain reaction (qRT-PCR)

Total RNA was isolated using TRIzol reagent (Roche, 49,916,800) and was extracted according to the manufacturer's protocols. cDNA was synthesized by Hiscript@ Q RT SuperMix (Vazyme, Nanjing, China, R223). qPCR was performed with Cham Q SYBR qPCR Master Mix (Vazyme, Q311) following the manufacturer's protocols. The mRNA expression levels were normalized to endogenous GAPDH and relative quantification was calculated using the 2-ΔΔCt method. The following primers were used: Collagen-I forward 5’-GTC CTG ATG GCA AAA CTG GC-3’, Collagen-I reverse 5’-CAG CAC CTT TAG GTC CAG GG-3’; MUC5AC forward 5’- TTA ACA TCC AGC TAC GCC GC-3’, MUC5AC reverse 5’- GAC CCC AGA CTG GCT GAA GG-3’; α-SMA forward 5’- ACT GGA CGA CAT GG A AAA G -3’, α-SMA reverse 5’- GAT CTC CAG CGT CCT GCA GA -3’; BMAL1 forward 5’- GTC TCA TTC TTC CAC GGG TA -3’, BMAL1 reverse 5’- GTG CTG CTG GCC ATT TAA GA -3’; GAPDH forward 5’-AGC CTC ACG CTA GTC AGT CG-3’, GAPDH reverse 5’-ATG ACC ACT CGG TTA TGC AT-3’.

### Immunofluorescence staining

Cells were seeded on coverslips prior to further treatment. Cells were fixed with 4% paraformaldehyde for 15–20 min and then blocked with 5% Bovine serum albumin (BSA, Servicebio, Wuhan, China, G5001-100G) in PBS for 1 h. Cells were incubated with primary antibodies against MUC5AC (1:200, Absin, abs126767) overnight. The next day, cells were treated with Cy3-labeled secondary antibody (1:200, Servicebio, GB21303) for 1 h. The nuclei in HBEs were stained with DAPI (Servicebio, G1012) for 10 min. The fluorescence images were performed by a laser-scanning confocal microscopy (LSM 780, Zeiss, Jena, Germany).

### Asthma animal model and treatments

Mice (C57BL/6 J) were purchased from VitalRiver (Beijing, China). *Bmal1* knockout (*Bmal1-KO*) mouse model was constructed by CRISPR/Cas9 gene editing. The sgRNA was designed and synthesized according to the *Bmal1* gene sequence, and then the synthesized sgRNA and Cas9 mRNA were injected into the fertilized mouse eggs. Cas9 nuclease and sgRNA bound to the mouse genome target sequence *Bmal1* and cut the double-stranded DNA, and then the *Bmal1* gene was subjected to frameshift mutation or fragment knockout by the non-homologous end joining repair. *Bmal1-KO* (*Bmal1*^−/−^) mice and *Bmal1-WT* (*Bmal1*^*wt/wt*^) mice were bred at Cyagen (Cyagen Biosciences, Guangzhou, China). All animal experiments involving mice were approved by the Institutional Animal Care and Use Committee of Tongji Medical College, Huazhong University of Science and Technology. In the acute asthma animal model, mice were treated with a suspension containing 10 mg OVA (Sigma, A5253, Grade II) and 2 mg aluminum hydroxide in 0.5 ml saline intraperitoneally on days 1, 7, and 10. Between days 21–27, mice were placed in an airtight container connected to an ultrasonic atomizer (Yuehua Medical Apparatus and Instruments, Guangzhou, China) and challenged with an aerosol of 3% OVA or saline (a flow rate of 2 ml/min) for 30 min a day. At the same time, mice were atomized with 0.1 g carbon particles dissolved in 50 mL 0.9% saline (for one hour each, twice times a day). Mice were euthanized on day 30. In the chronic asthma animal model, mice were injected intraperitoneally with 10 mg ovalbumin and 2 mg aluminum hydroxide in 0.5 ml saline on days 1 and 10. From day 14. mice were placed in the airtight container described above and inhaled aerosolized 3% OVA or saline for 30 min a day (three times a week). Meanwhile, the experimental groups were challenged by nebulized inhalation of 0.1 g carbon particles dissolved in 0.9% saline or PM2.5 dissolved in 0.9% saline (twice a day, five times a week). The control groups underwent aerosol inhalation with the same volume of saline instead.

### Bronchoalveolar lavage fluid (BALF) and cell counting

Mice (male C57BL/6 J) were terminally anaesthetized. The neck skin of mice was cut open to expose the trachea. Then a cannula was inserted into the airways to obtain BALF of the whole lung. After lavage three times with 1.2 ml PBS, BALF was centrifuged to pellet by centrifuging at 1500 r/min for 10 min. BAL cells were re-suspended and harvested with PBS and then determined using the Hemocytometer.

### Enzyme-linked immunosorbent assay (ELISA)

Enzyme-linked immunosorbent assay (ELISA) kits were used to measure BALF levels of IL-4 (Mouse IL-4 ELISA Kit; Dakewe), IL-5 (Mouse IL-5 ELISA Kit; Dakewe), IL6 (Mouse IL-6 ELISA Kit; Dakewe), IL-13 (Mouse IL-13 ELISA Kit; Dakewe) and MUC5AC (Mouse MUC5AC ELISA Kit, Dakewe). BALF were collected as described above and stored at − 20 °C until the assay was performed using the ELISA kits. Subsequent steps were carried out following the manufacturer’s protocol.

### Transmission electron microscopy

Transmission electron microscopy was performed to assess microstructural changes of HBEs. The cells were fixed with 2.5% glutaraldehyde at 4 °C overnight and post-fixed in 1% osmium tetroxide for 30 min. After being dehydrated in a graded series of ethanol baths, the cells were embedded in the epoxy resin media. The resin blocks were then cut into ultrathin sections, stained with uranyl acetate and lead citrate, and viewed by TEM (Tecnai G^2^ 20 TWIN, FEI Company, Hillsboro, OR, USA).

### Immunohistochemistry (IHC)

Mouse lung tissues were fixed with 4% paraformaldehyde and embedded in paraffin for IHC. Paraformaldehyde-fixed, paraffin-embedded Sects. (5 μm) were dewaxed, dehydrated and rehydrated successively and stained by Masson, H&E, PAS and MUC5AC antibody, respectively. After staining, the lung sections were examined by light microscopy connected with a digital camera. The number of PAS-positive cells in airways was counted by light microscopy (× 400 magnification), and the results are expressed as the percentage of PAS + epithelial cells out of the total number of epithelial cells in the airway. Masson trichrome staining was used to evaluate collagen deposition. Deposition of collagen was quantified as a percentage of surface area stained with ImageJ software.

### RNA sequencing and analysis

RNA-Seq was performed by CapitalBio Technology on an Illumina NovaSeq sequencer (Illumina). Total RNA of HBEs was isolated from 3 independent preparations using TRIzol reagent following the manufacturer’s protocol. The sequencing quality was evaluated utilizing FastQC and low-quality data were removed using NGSQC[49]. DESeq algorithm was applied to identify the DEGs between the siRNA-NC group and siRNA-BMAL1 group. Parameters for classifying significantly DEGs are as follows: |log2FC|> 1 and p < 0.05, FC: the fold change of expressions. The functional annotation database of DEGs was carried out using multiple databases, including Ensemble, National Center for Biotechnology Information, Uniprot, GO, and Kyoto Encyclopedia of Genes and Genome (KEGG).

### Co-immunoprecipitation (Co-IP) assay

Co-immunoprecipitation experiments were performed following manufacturers protocols (MCE, Shanghai, China, HY-K0202). Cells were harvested and lysed in IP buffer supplemented with protease inhibitor at 4 °C for one hour. The lysed cells were mixed with the appropriate antibodies and incubated at 4 °C overnight. The next day, cell lysates containing the antibody were incubated with protein A/G magnetic beads (MCE, Shanghai, China) with gentle shaking for 1–2 h at 4 °C. Magnetic beads were separated from cell lysates in the magnetic stand and washed four times with lysis buffer. 25–50 µl 2 × SDS-PAGE Loading Buffer was added into the Protein A/G Magnetic Beads-Ab-Ag complex. The supernatant was collected after the mixture was heated at 95 °C for 5 min. Western blot was used to detect cross-correlations of the related protein in the whole-cell lysate.

### Statistical analysis

In experiments using the cell lines (HBEs, HEK293T), the experimental group and the control group had the same number of passage and similar density to ensure that the experimental conditions are consistent. In animal studies, mice were randomly allocated to the experimental or control group. Results were statistically analyzed using unpaired *t-*tests, one-way ANOVA test or two-way ANOVA test with Prism software version 8. All data are represented as the mean ± SEM. A *p*-value < 0.05 was considered significant.

## Supplementary Information

**Additional file 1:** Supplementary figures.

## Data Availability

All data and materials are included in the manuscript.

## Abbreviations

PM2.5: Fine particulate matter
α-SMA: Alpha-smooth muscle actin
ATG5: Autophagy-related gene 5
LC3B-II: Type II form of microtubule-associated protein 1 light chain 3 beta
Co-IP: Co-immunoprecipitation
