# Supplementary material for: BMAL1/p53 mediating bronchial epithelial cell autophagy contributes to PM2.5-aggravated asthma
Source: Cell Commun Signal. 2023 Feb 20;21:39. doi: 10.1186/s12964-023-01057-9 (PMC9940367; doi:10.1186/s12964-023-01057-9)
Supplement: Supplementary file 2 — Additional file 1: Supplementary figures. [file 12964_2023_1057_MOESM2_ESM.pdf]

Online supplementary materials

Title: BMAL1/p53 mediating bronchial epithelial cell autophagy contributes to PM2.5-aggravated asthma

Authors: Shuai-Jun Chen *et al*

Contents

Fig. S1. PM2.5 exposure aggravated airway remodeling in mouse chronic asthma model

Fig. S2. Carbon particles exposure exacerbated asthma manifestation in mouse acute model

Fig. S3. Carbon particles/PM2.5 exposure induced up-regulation of collagen-I, α-SMA and MUC5AC in bronchial epithelial cells in vitro

Fig. S4. Verification of BMAL1 overexpression or knockdown in HBEs and knockout in mice

Fig. S5. mRNA sequencing of human HBEs after BMAL1 depletion

Fig. S6. Carbon particles/PM2.5 induced autophagy in bronchial epithelial cells

Fig. S7. Inhibition of autophagy by HCQ reversed carbon/PM2.5 induced collagen-I, α-SMA, fibronectin expression and cell autophagy in bronchial epithelial cells in vitro

Fig. S8. Inhibition of autophagy with ATG5-siRNA reversed carbon/PM2.5 induced collagen-I, fibronectin expression and cell autophagy in bronchial epithelial cells in vitro

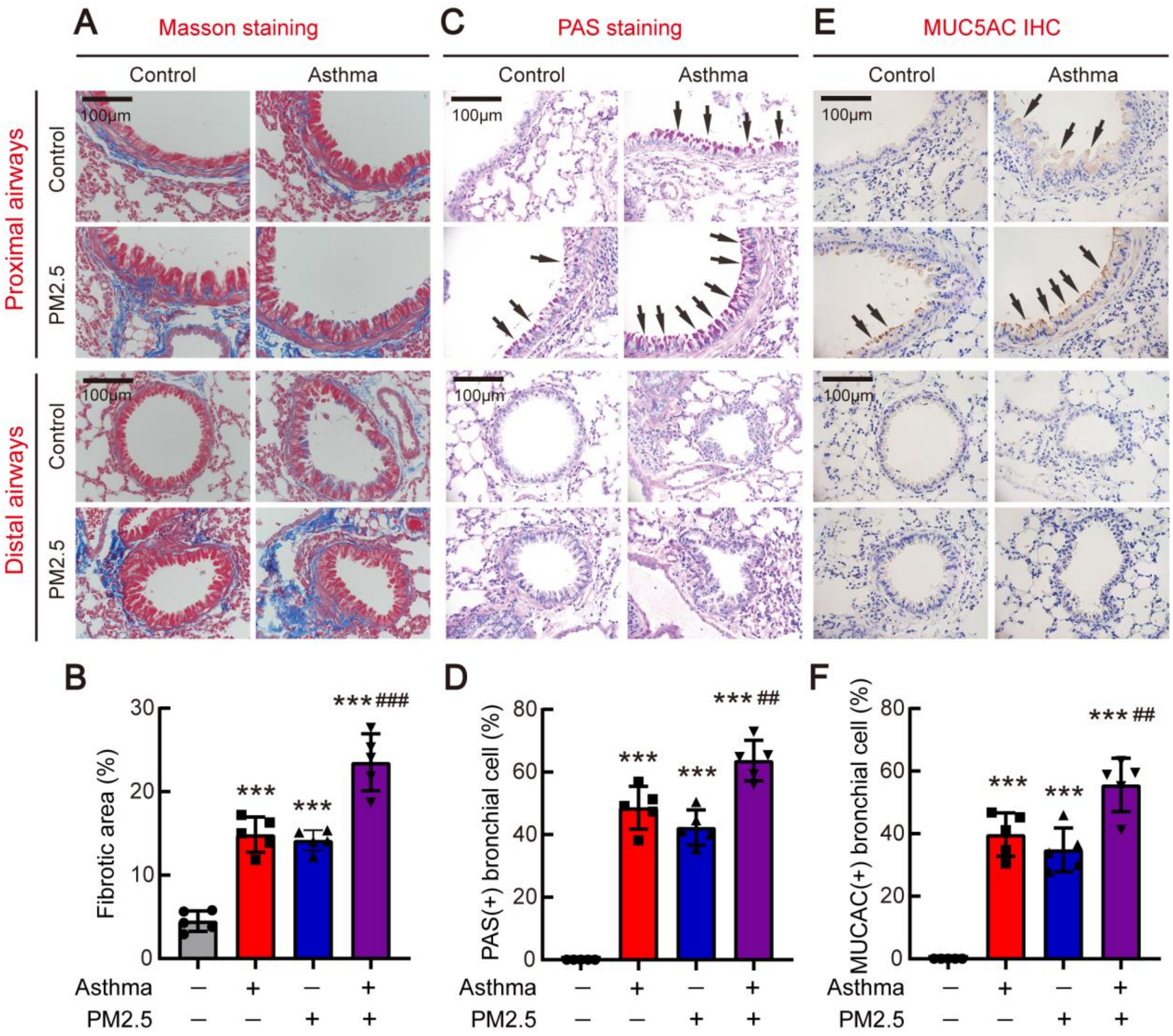

Fig. S1. PM2.5 exposure aggravated airway remodeling in mouse chronic asthma model. Mice were sensitized with intraperitoneal injection of 10 mg OVA, and then challenged by repeated aerosol challenges containing 3% OVA and PM2.5 over a 6-week period as described in the Methods. Lungs were harvested at day 60. Masson staining (A), PAS staining (C), and immunohistochemistry (IHC) staining of MUC5AC (E) was performed. Top two panels: proximal airway; Bottom two panels: distal airway. Original magnification, ×400. (B) Collagen deposition area (%) of proximal airways in each group (n=5). (D) The quantification histograms of PAS staining in proximal airways (n=5). (F) The quantification histograms of IHC MUC5AC staining in proximal airways (n=5). Data are shown as mean ±

SEM of n individual experiments. $^*P < 0.05$, $^{**}P < 0.01$, $^{***}P < 0.001$ (versus control), $^\#P < 0.05$, $^{\#\#}P < 0.01$, $^{\#\#\#}P < 0.001$ (versus asthma). *P* values were determined by one-way ANOVA followed by the Bonferroni's test.

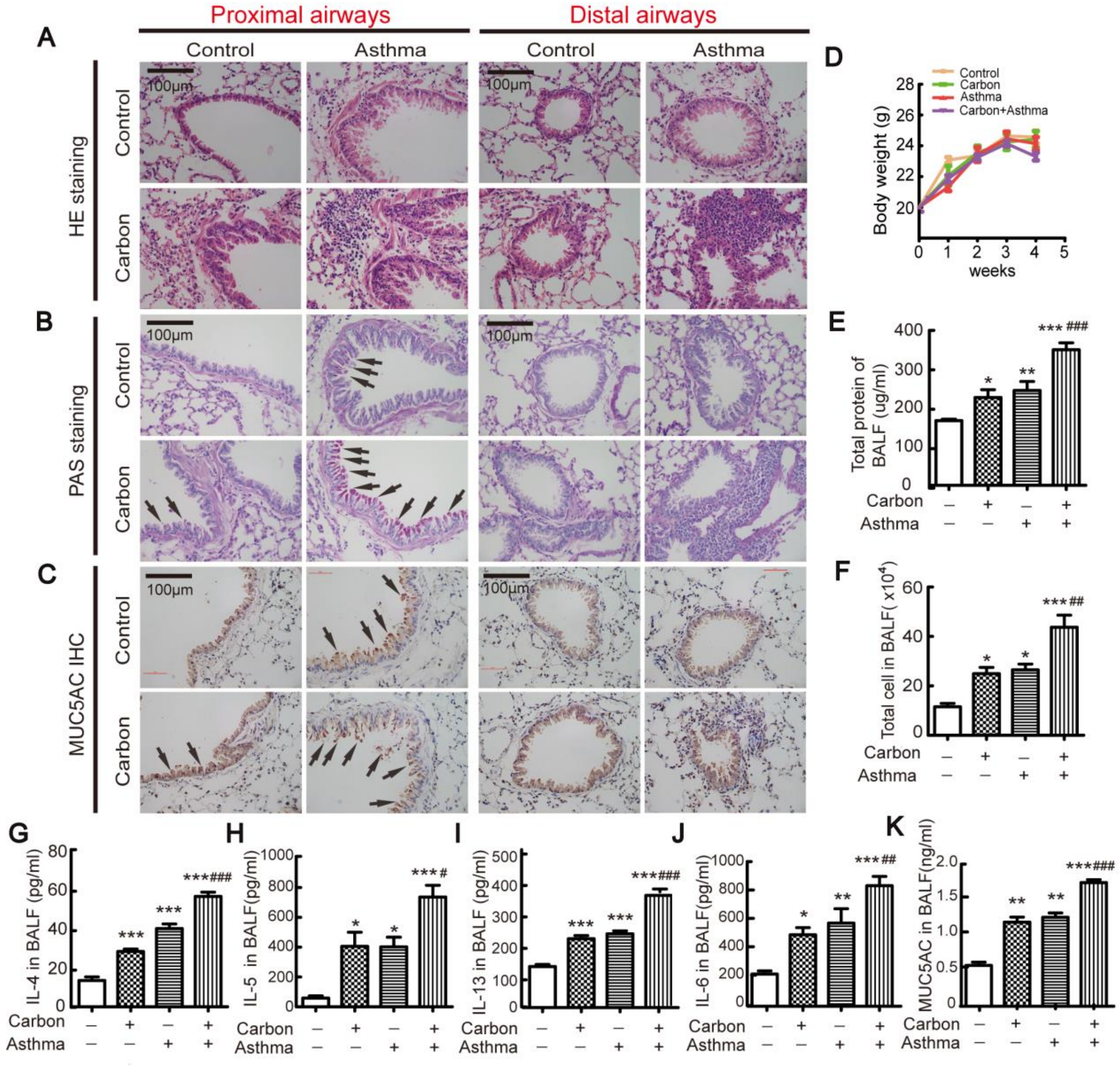

Fig. S2. Carbon particles exposure exacerbated asthma manifestation in mouse acute model. C57BL/6 J mice were sensitized and challenged with OVA as described in the Methods. Between days 21-27, aerosol challenges containing either 3% OVA or saline were given to mice for 30 min a day. At the same time, mice were atomized with 0.1g carbon particles dissolved in 50mL 0.9% saline twice daily. (A-C) Representative images of lung sections with Masson staining, PAS staining and immunohistochemistry (IHC) staining for MUC5AC, respectively. Original magnification, ×400. (D) Mean body weight changes of mice. (E) The concentrations of total protein in BALF were assayed by BCA, n=7. (F) The

numbers of total inflammatory cells in BALF were counted by Hemocytometer. n=6 (control group and carbon particles group), n=8 (asthma group), n=7 (carbon particles + asthma group). IL-4 (G), IL-5 (H), IL-13 (I), IL-6 (J), and MUC5AC (K) protein expression in BALF detected by ELISA. Data are expressed as mean ± SEM. **P* < 0.05, ***P* < 0.01, ****P* < 0.001 (versus control), #*P* < 0.05, ##*P* < 0.01, ###*P* < 0.001 (versus asthma). *P* values were determined by one-way ANOVA.

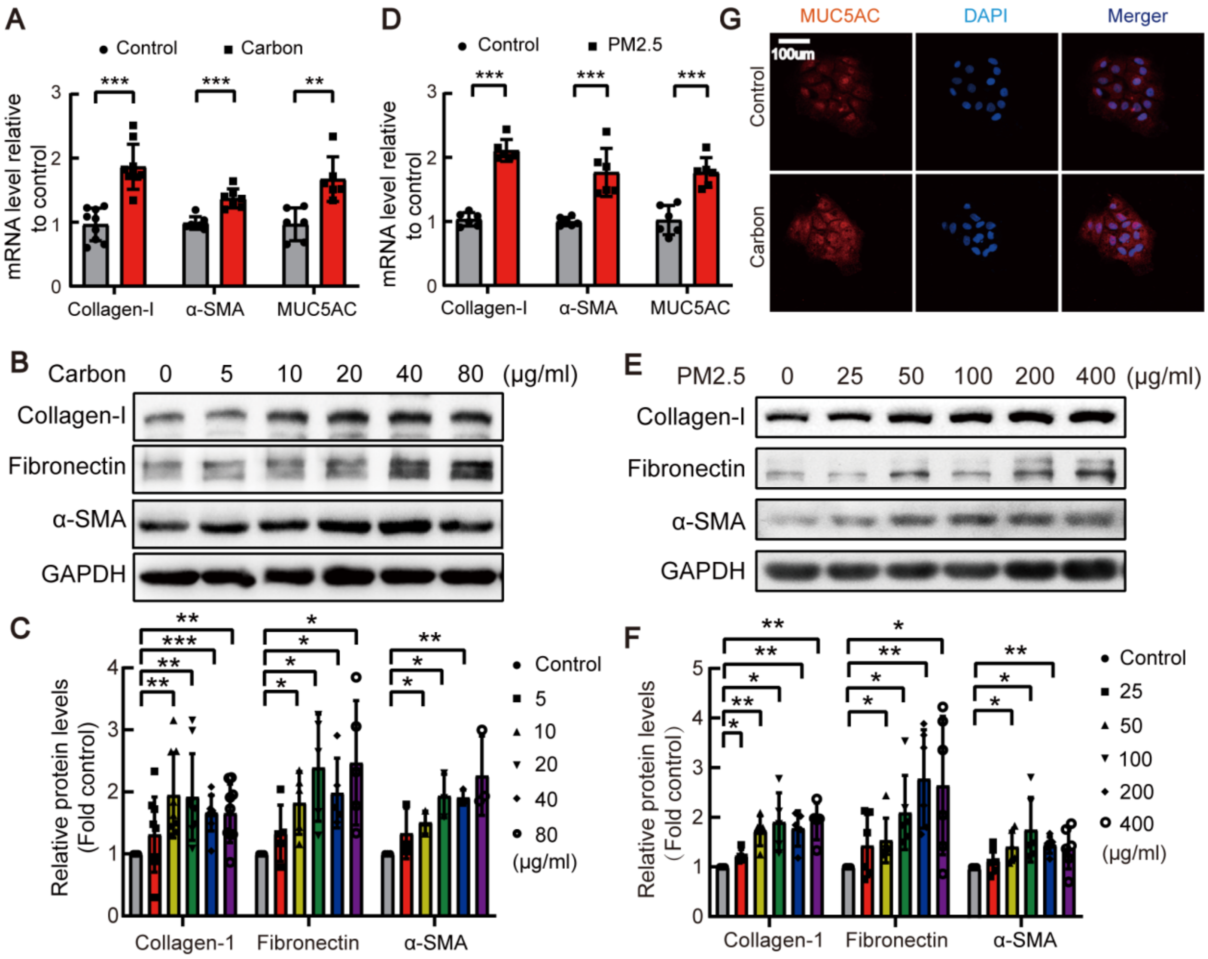

Fig. S3. Carbon particles/PM2.5 exposure induced up-regulation of collagen-I, α-SMA and MUC5AC in bronchial epithelial cells in vitro. (A, D) HBEs were treated with carbon particles or PM2.5 for 24 h, mRNA levels of collagen-I, α-SMA and MUC5AC were investigated by quantitative RT-PCR (RT-qPCR) and normalized to the housekeeping gene glyceraldehyde-3-phosphate dehydrogenase (GAPDH). n=6. (B, C, E-G) HBEs were treated with carbon particles or PM2.5 concentration gradients for 48 h, after which expressions of intracellular levels of fibronectin, collagen-I, α-SMA and MUC5AC were investigated by Western blotting or immunofluorescence (IF) assay. (B, E) Representative western blots. (C, F) Changes in relative density of blots according to B, E. (C): n=8 (collagen-I), n=5 (fibronectin) and n=3 (α-SMA). (F): n=5 (collagen-I), n=6 (fibronectin, α-SMA). (G) Representative immunofluorescence staining images of MUC5AC. Data are mean ± SEM of

n individual experiments, *$P < 0.05$, **$P < 0.01$, ***$P < 0.001$ (between indicated conditions). *P* values were determined by one-way ANOVA followed by the Dunnett's test (C, F) or unpair Student's *t*-test (A, D).

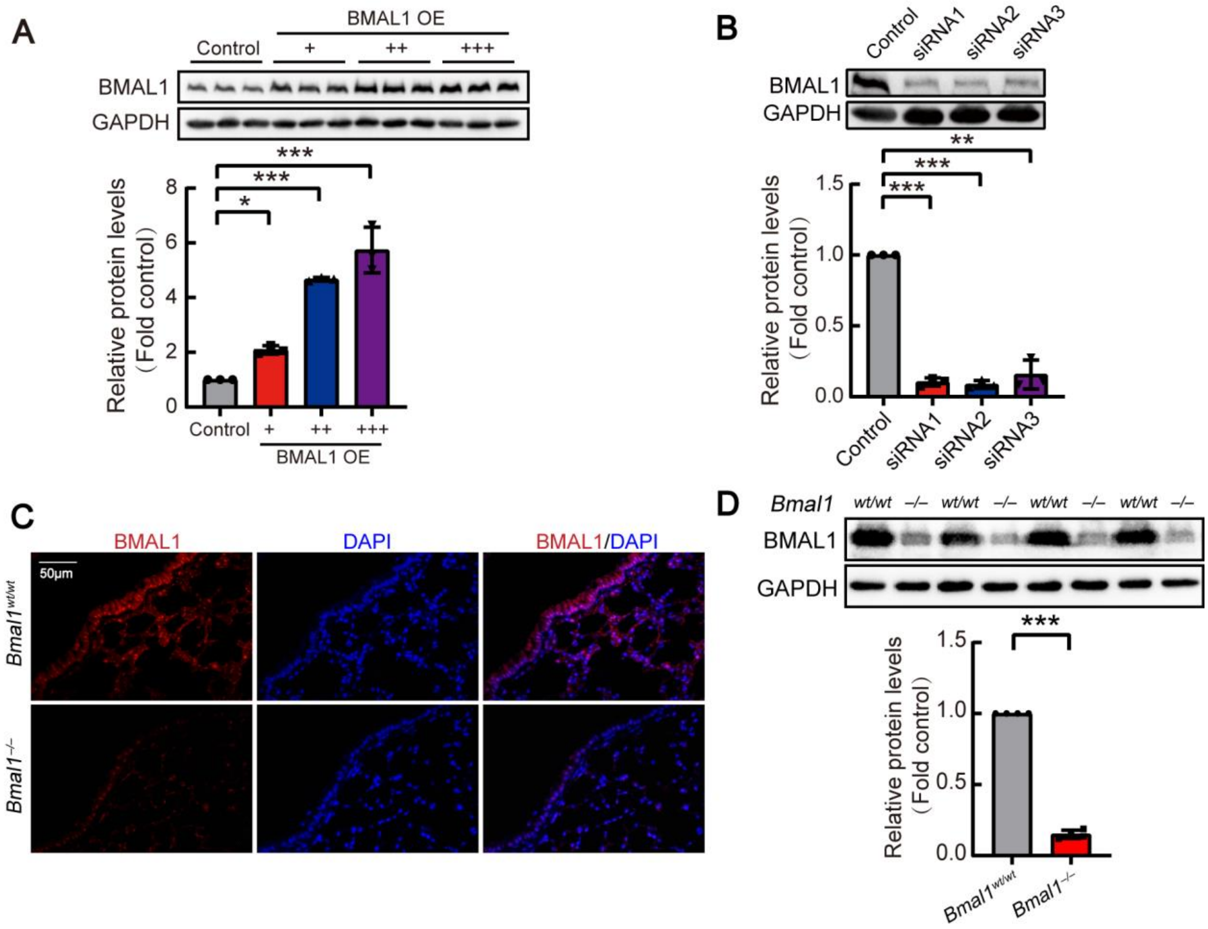

Fig. S4. Verification of BMAL1 overexpression or knockdown in HBEs and knockout in mice. (A) HBEs were seeded into 3.5 cm dishes and transduced with control (Vector) or BMAL1 overexpressing (BMAL1 OE) vectors for 72 h (+, ++, and +++ separately represented 2 μg, 4 μg, and 8 μg BMAL1 OE vector transduced in HBEs). BMAL1 protein levels were investigated by Western blot. n=3. (B) Representative bolt images and bar graphs to display changes of BMAL1 protein in HBEs treated with control siRNA or specific siRNA against BMAL1 for 48 h. n=3. (C) Representative images of BMAL1 staining of lung tissue sections. Scale bars: 50 μm. (D) Western blot images and analysis of BMAL1 knockout efficiency in lung tissue, n=4. Data are shown as mean ± SEM of n individual experiments. *P < 0.05, **P < 0.01, ***P < 0.001 between indicated conditions (Student's *t*-test).

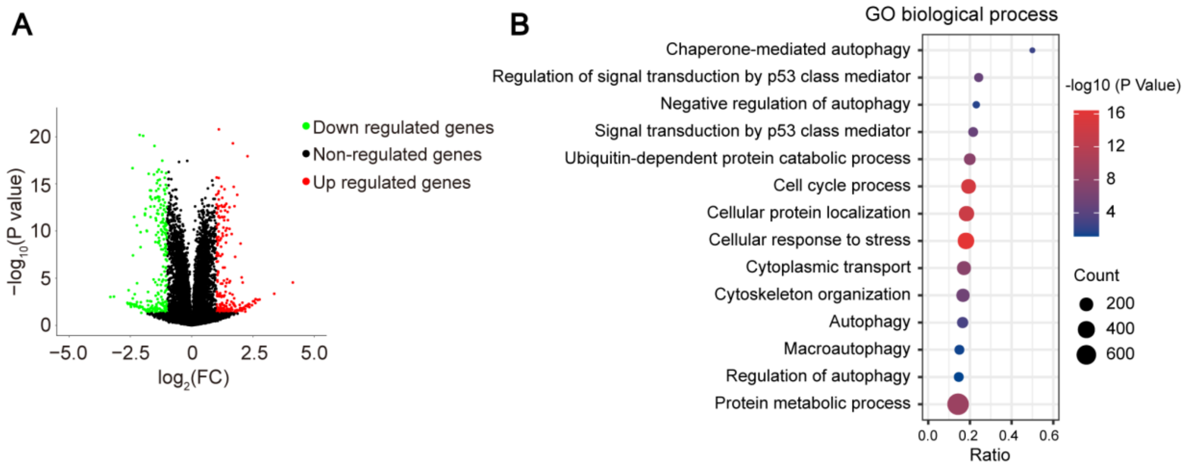

Fig. S5. mRNA sequencing of human HBEs after BMAL1 depletion. (A) The volcano-plot of differentially expressed genes. *P* value < 0.05 and fold change > 2 were set as restrictive conditions to identify the differentially expressed genes. (B) GO biological process (BP) enrichment analysis showed differentially expressed genes that related to different GO terms.

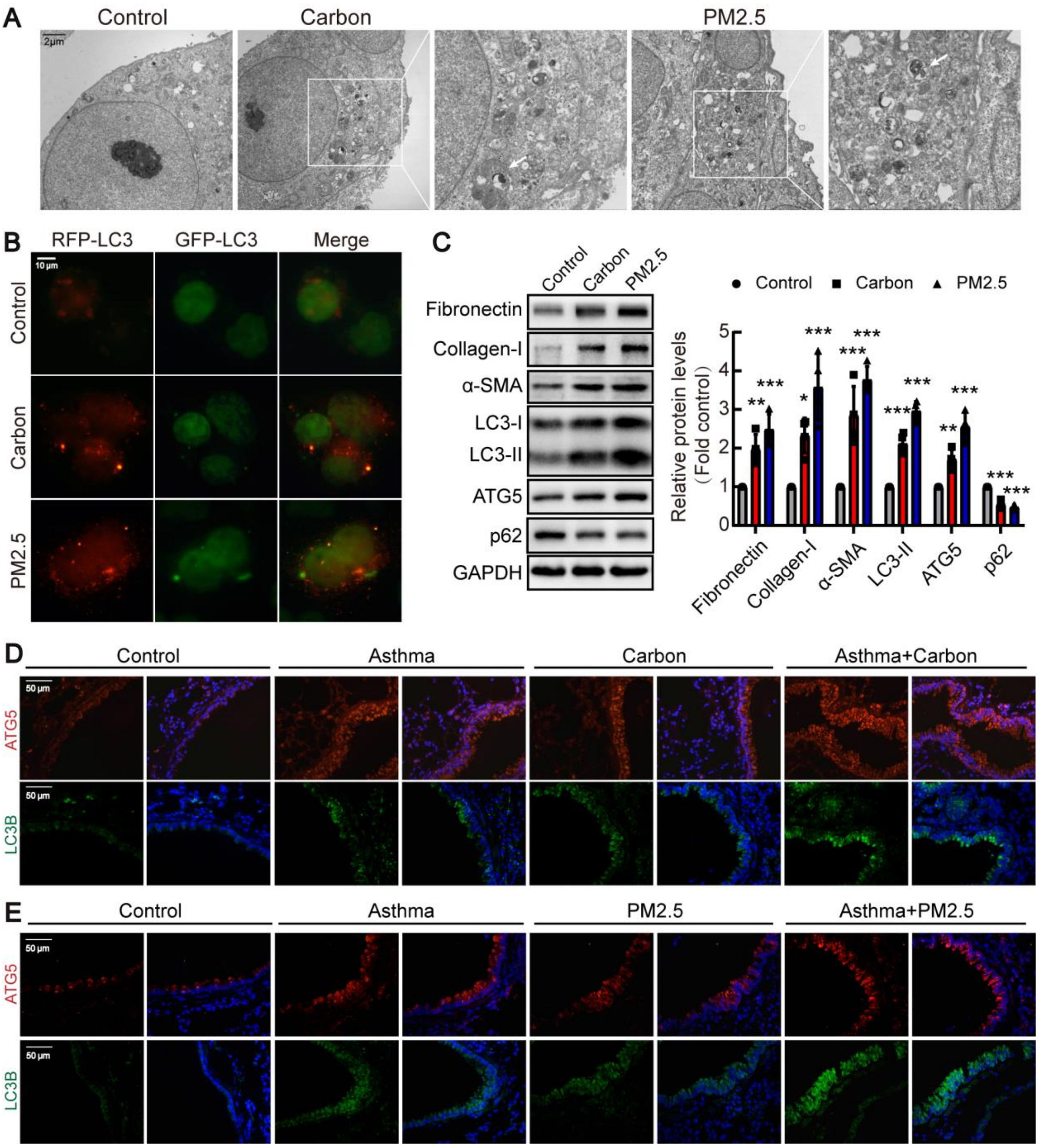

Fig. S6. Carbon particles/PM2.5 induced autophagy in bronchial epithelial cells. (A-C) After treated with carbon particles/PM2.5 for 24 h**,** the changes of autophagy in airway epithelial cells were detected. (A) Electron microscopy images of autophagosomes in HBEs; arrowheads indicate autophagosomes. (B) Representative images of GFP-LC3 and RFP-LC3 fluorescence staining. Scale bar: 10 μm. (C) After treated with carbon particles/PM2.5 for 24 h, representative western blots and protein changes of fibronectin, collagen-I, α-SMA, LC3-II,

ATG6, and p62 in HBEs, n=3. (D) The OVA and carbon particles-induced asthma model. The immunofluorescence pictures of ATG5 and LC3B. Scale bar: 50 μm. (E) The OVA and PM2.5-induced asthma model. The immunofluorescence pictures of ATG5 and LC3B. Scale bar: 50 μm. Data are mean ± SEM of n individual experiments, $^{*}P < 0.05$, $^{**}P < 0.01$, $^{***}P < 0.001$ between indicated conditions (Student's *t*-test).

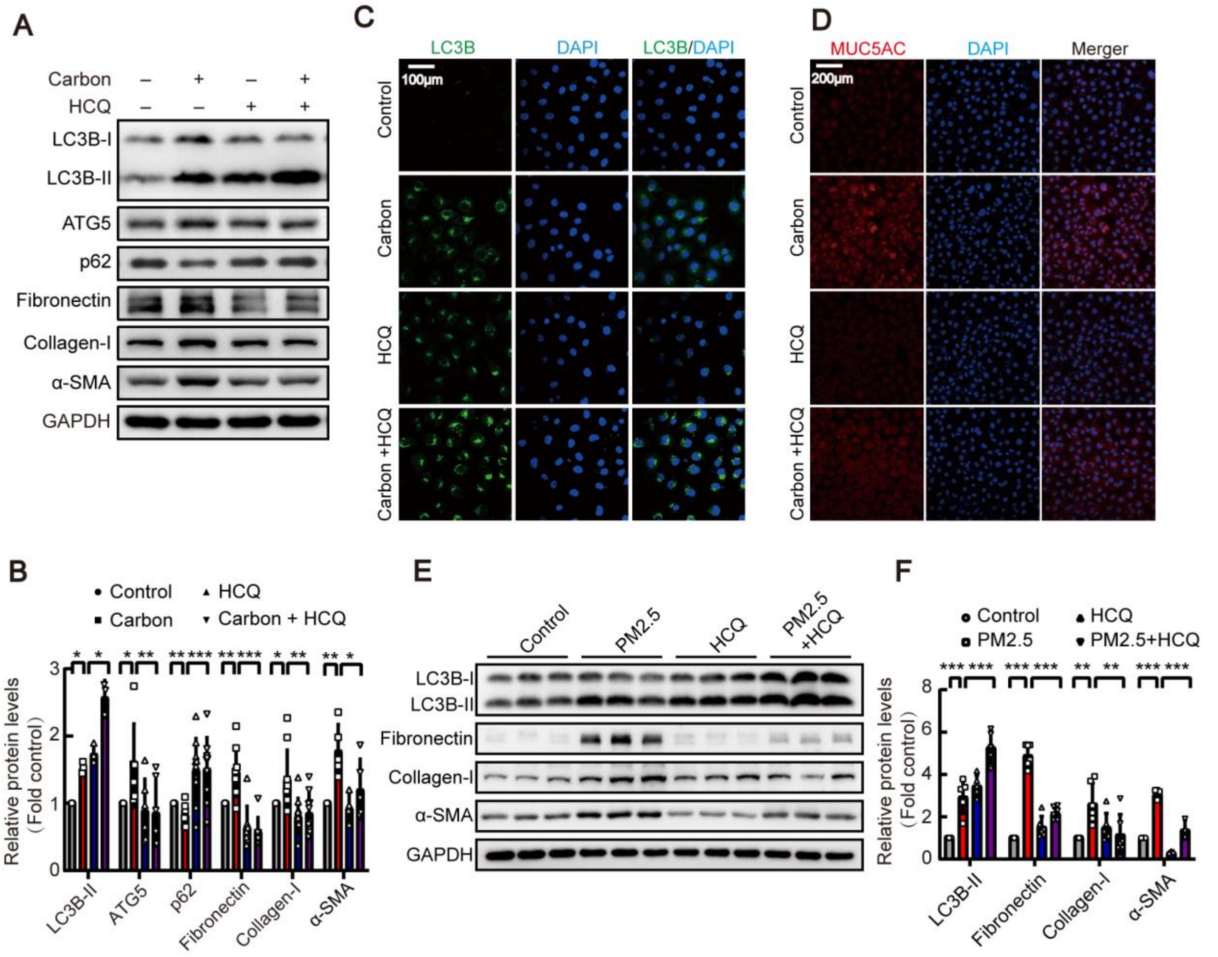

Fig. S7. Inhibition of autophagy by HCQ reversed carbon/PM2.5 induced collagen-I, α-SMA, fibronectin expression in bronchial epithelial cells in vitro. HBEs were treated with HCQ and carbon particles (20 μg/ml) or PM2.5 (200 μg/ml) for 48 h. The levels of related protein and mRNA were measured by western blotting, RT-qPCR or immunofluorescence staining. (A) Representative images of Western blots of LC3B-II, ATG5, collagen-I, fibronectin, p62, and α-SMA were shown. (B) Changes in relative density of LC3B-II, ATG5, fibronectin, collagen-I, p62, and α-SMA to GAPDH were presented. n=4 (LC3B-II), n=6 (ATG5), n=8 (fibronectin), n=10 (collagen-I, p62) and n=5 (α-SMA). (C) Representative immunofluorescence staining images of LC3B-II. Original magnification, ×400. (D) Representative immunofluorescence staining images of MUC5AC. Original magnification, ×200. (E) Representative images of Western blots of LC3B-II, collagen-I,

fibronectin and α-SMA were shown. (F) Changes in relative density of LC3B-II, fibronectin, collagen-I and α-SMA to GAPDH were presented. n= 6 (fibronectin, collagen-I), n=5 (LC3B-II), n=3 (α-SMA). Data are mean ± SEM of n individual experiments, **P* <0.05, ***P* < 0.01, ****P* < 0.001 between indicated conditions (One-way ANOVA followed by the Bonferroni's test).

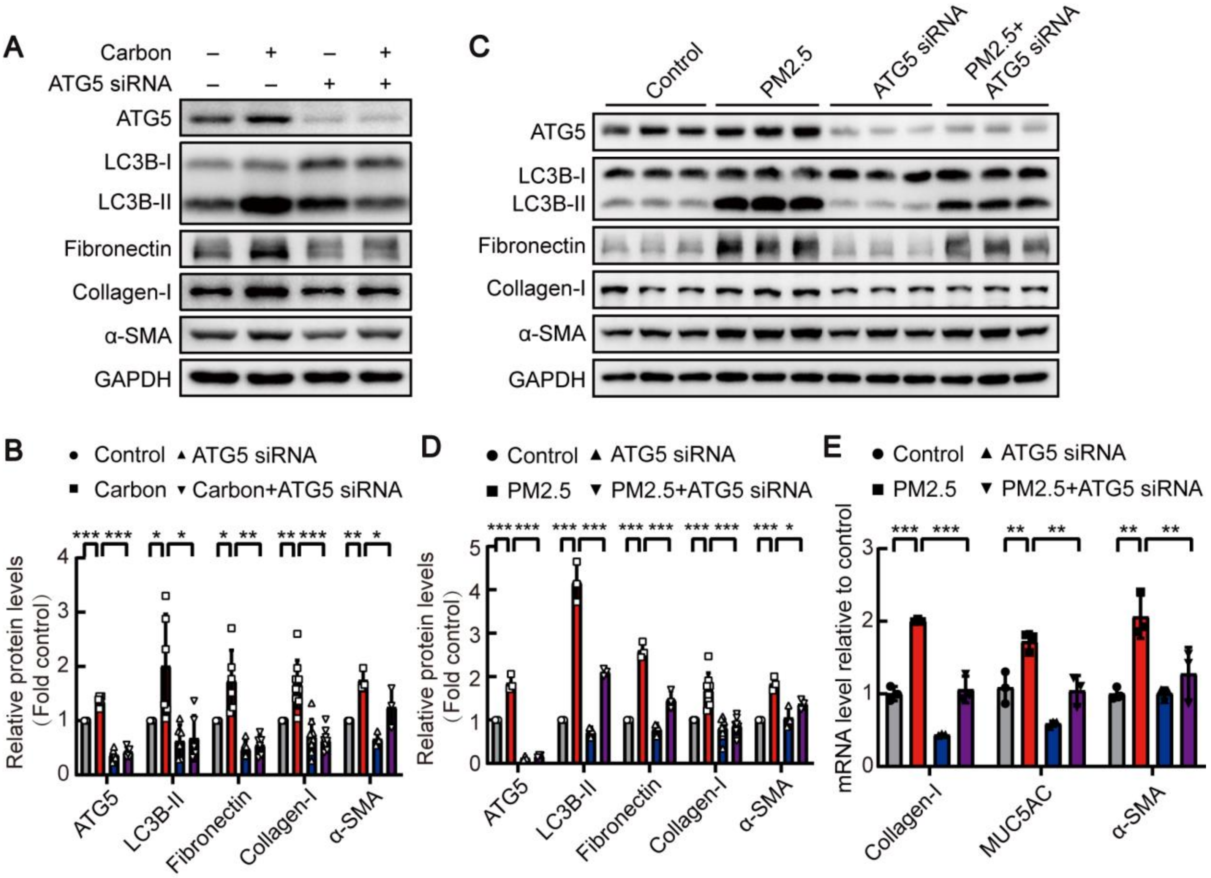

Fig. S8. Inhibition of autophagy with ATG5-siRNA reversed carbon particles/PM2.5 induced collagen-I, fibronectin expression and cell autophagy in bronchial epithelial cells *in vitro*. HBEs were incubated for 48 h after transfection with control or specific siRNA against ATG5 and treated with carbon particles (20 μg/ml) or PM2.5 (200 μg/ml) for 48 h prior to harvest of the cell lysate, after which intracellular ATG5, LC3B-II, fibronectin, collagen-I and α-SMA protein content was measured by western blotting (A, C). Changes in relative density of ATG5, LC3B-II, fibronectin, collagen-I and α-SMA to GAPDH were presented (B, D). mRNA expression of collagen-I, MUC5AC and α-SMA was measured by RT-qPCR and normalized to GAPDH (E). Data are mean ± SEM of n individual experiments. B: n=6 (fibronectin, ATG5, LC3B-II), n=9 (collagen-I), n=3 (α-SMA); D: n=3 (ATG5, LC3B-II, fibronectin, α-SMA), n=8 (collagen-I); E: n=3. *P <0.05, **P < 0.01, ***P < 0.001 between indicated conditions (One-way ANOVA followed by the Bonferroni's test).
